# Spatially Self‐Organized Three‐Dimensional Neural Concentroid as a Novel Reductionist Humanized Model to Study Neurovascular Development

**DOI:** 10.1002/advs.202304421

**Published:** 2023-11-30

**Authors:** Yoke Chin Chai, San Kit To, Susan Simorgh, Samantha Zaunz, YingLi Zhu, Karan Ahuja, Alix Lemaitre, Roya Ramezankhani, Bernard K. van der Veer, Keimpe Wierda, Stefaan Verhulst, Leo A. van Grunsven, Vincent Pasque, Catherine Verfaillie

**Affiliations:** ^1^ Stem Cell Institute Leuven Department of Development and Regeneration KU Leuven, O&N4, Herestraat 49 Leuven 3000 Belgium; ^2^ Stem Cell Institute Leuven Department of Development and Regeneration Leuven Institute for Single Cell Omics (LISCO) KU Leuven, O&N4, Herestraat 49 Leuven 3000 Belgium; ^3^ Laboratory for Stem Cell and Developmental Epigenetics Department of Development and Regeneration KU Leuven, O&N4, Herestraat 49 Leuven 3000 Belgium; ^4^ Electrophysiology Expert Unit VIB‐KU Leuven Center for Brain & Disease Research Leuven 3000 Belgium; ^5^ Liver Cell Biology Research Group Vrije Universiteit Brussel (VUB) Brussels 1090 Belgium

**Keywords:** blood‐brain barrier, brain organoids, neurovascular, vascularization

## Abstract

Although human pluripotent stem cell (PSC)‐derived brain organoids have enabled researchers to gain insight into human brain development and disease, these organoids contain solely ectodermal cells and are not vascularized as occurs during brain development. Here it is created less complex and more homogenous large neural constructs starting from PSC‐derived neuroprogenitor cells (NPC), by fusing small NPC spheroids into so‐called concentroids. Such concentroids consisted of a pro‐angiogenic core, containing neuronal and outer radial glia cells, surrounded by an astroglia‐dense outer layer. Incorporating PSC‐derived endothelial cells (EC) around and/or in the concentroids promoted vascularization, accompanied by differential outgrowth and differentiation of neuronal and astroglia cells, as well as the development of ectodermal‐derived pericyte‐like mural cells co‐localizing with EC networks. Single nucleus transcriptomic analysis revealed an enhanced neural cell subtype maturation and diversity in EC‐containing concentroids, which better resemble the fetal human brain compared to classical organoids or NPC‐only concentroids. This PSC‐derived “vascularized” concentroid brain model will facilitate the study of neurovascular/blood‐brain barrier development, neural cell migration, and the development of effective in vitro vascularization strategies of brain mimics.

## Graphical abstract

A less complex and spatially self‐organized homogeneous large neural construct, named Neural Concentroid, is reported. The concentroid consists of a pro‐angiogenic core, contains neuronal and outer radial glia cells, and is surrounded by an astroglia‐dense outer layer. Co‐culture with iPSC‐derived endothelial cells generates vascularized concentroids, which represents a novel reductionist model that allows the study of neurovascular interactions and maturation.

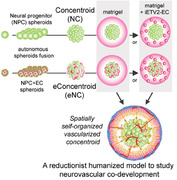

## 1 Introduction

Recent advances in brain organoid technology have revolutionized the field of modern neuroscience. Scientists can now grow 3D human brain‐like structures from pluripotent stem cells (PSCs) to study neurodevelopment and neurological disorders in vitro.^[^ ^1^, ^2^, ^3^, ^4^ ^]^ These brain mimics possess many of the salient neural cytoarchitecture with transcriptional and epigenetic landscapes reminiscent of early to mid‐fetal brain development,^[^ ^5^, ^6^, ^7^ ^]^ and in longer‐term culture induces neural maturation that matches key early postnatal transitions.^[^ ^8^ ^]^ Fusion of different organoids resembling different brain regions also allow scientist to create models with different brain region‐specificities^[^ ^9^, ^10^, ^11^, ^12^, ^13^ ^]^ or cortico‐motor functionality.^[^ ^14^ ^]^ Although brain organoids contain a multitude of cortical cells,^[^ ^7^ ^]^ further technological improvement is needed as they form for instance redundant ventricular zones (VZs),^[^ ^15^ ^]^ and different cellular makeup and organization even when made simultaneously from the same PSC population.^[^ ^16^ ^]^ Uniformity of organoid cytoarchitecture is necessary to allow predictive translational research outcomes which otherwise prevents robust readouts of cellular responses. Hence, integrating bioengineering approaches, among others, to generate more uniform 3D neural models efficiently remains highly demanded.^[^ ^17^, ^18^, ^19^ ^]^

Importantly, brain organoids do not contain vessels. Aside from their importance for nutrient‐waste exchange in the brain,^[^ ^20^ ^]^ there is abundant evidence that vasculature co‐development plays a crucial role in proper developmental neurogenesis,^[^ ^21^ ^]^ brain architecture, and plasticity.^[^ ^22^, ^23^ ^]^ The early cerebral cortex is avascular as the peri‐neural vascular plexus only starts to sprout radially into the cortical pre‐plate at 9–9.5 weeks of gestation under a gradient of vascular endothelial growth factor (VEGF).^[^ ^24^, ^25^, ^26^ ^]^ Brain organoids mimic early neural tube formation without need for vasculature.^[^ ^27^ ^]^ However, at later stages, these organoids fail to attain mature brain cell composition, thought to be at least in part due to the lack of co‐developing vasculature. Various in vitro vascularization strategies have been developed, including co‐culture with endothelial cells (ECs),^[^ ^28^, ^29^, ^30^ ^]^ fusion with vessel organoids,^[^ ^31^ ^]^ or creation of perfusable microvasculature using microfluidic devices.^[^ ^32^, ^33^ ^]^ Of note, when vascularized, the VZs within typical brain organoids are disrupted, in contrast to what occurs during fetal brain development.

In this study, we opted to create the first simpler, large pro‐angiogenic homogeneous cortical mimics to evaluate the effect of in vitro vascularization on neural development. We therefore created large but structurally uniform so‐called neural concentroids (NC) by fusing PSC‐NPC‐derived spheroids, with a large hypoxic core. This model enabled us to assess the impact of ECs (either incorporated within and/or surrounding the concentroid) on both neuronal and glial migratory behavior as well as vessel ingrowth into the concentroid under the influence of a VEGF gradient originating from the pro‐angiogenic core. Additionally, single nucleus transcriptomic analysis demonstrated how vascularization affected neuronal and glial cell maturation.

## 2 Results

### 2.1 Spatially Self‐Organized Neural Concentroids have Forebrain Identity, Promoting Neuronal Subtype Specification and Astroglial Maturation

We produced homogenous batches of neural spheroids in a medium‐throughput manner by seeding pre‐differentiated NPCs obtained following dual‐SMAD inhibition^[^ ^34^, ^35^ ^]^ on 3D‐printed agarose microwell culture inserts (Figure S1A,B, Supporting Information). Cell viability was confirmed by live cell fluorescence imaging of the spheroids based on the built‐in tdTomato transgene (Figure S1C, Supporting Information). Spheroid growth was assessed by measuring the spheroid diameter (Figure S1D, Supporting Information). Expression of key markers for different neural cell types over 105 days of culture was assessed by RT‐qPCR and immunofluorescence staining at defined time points. This demonstrated that neural spheroids consisted initially of neurons (high expression of TUJ1 at early and MAP2 at later time points; low expression of upper and deep layer cortical neuronal markers *TBR2*, *TBR1*, *CTIP2*, and *SATB2*; Figure S1E,F, Supporting Information) and radial glial cells (Figure S1G, Supporting Information). At later time points, astroglial cells also were present^[^ ^36^, ^37^ ^]^ (consistently high *SOX9* expression with progressive upregulation of canonical astrocyte markers *GFAP*, *AQP4*, and *ALDH1L1* ^[^ ^38^ ^]^; Figure S1H, Supporting Information)). Expression of the oligodendrocyte progenitor transcript, *SOX10*, remained low (Figure S1I, Supporting Information).^[^ ^39^ ^]^

**Figure 1:**
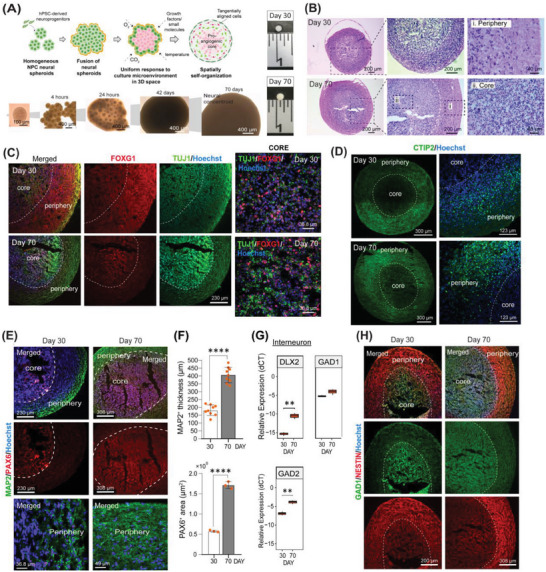
Neuronal subtype specification in spatially self‐organized cytoarchitecture. A) Schematics showing the concept of generating spatially self‐organized neural concentroid from hPSC‐derived neuroprogenitor cells (NPCs), and the corresponding images of neural spheroids, fused spheroids (within 4 to 24 h), and concentroids at different stages. B) Histology of concentroid mid‐cryosection on days 30 and 70 showed distinctive zonation with tangential aligned cells at the periphery and dense cell core (H&E staining). C) Immunostaining showing the spatial distribution of FOXG1^+^ and TUJ1^+^ cells within the concentroids on days 30 and 70. D,E) Immunostaining for CTIP2 (periphery), PAX6 (core) and MAP2 (periphery) within the concentroid. F) Quantification of area occupied by MAP2^+^ (*n* = 3, three measurements per sample) and PAX6^+^ (*n* = 3) cells in periphery and core of concentroids over time (one‐way ANOVA with Tukey's multiple comparisons test). G) Boxplots of the RT‐qPCR results for *DLX2, GAD1* and *GAD2* (*n* = 3, duplicate measurements). H) Immunostaining for GAD1 and NESTIN in the concentroids on days 30 and 70. All data shown are mean ± SD: unpaired *t*‐test: **p < 0.01, ****p < 0.0001. Nuclei were counterstained with Hoechst.

We then generated neural concentroids (NC) by fusing ≈130 NPC spheroids per NC on an orbital shaker for 30 days in the presence of BDNF‐ and NT3‐supplemented neural maintenance medium (NMM) to promote neuronal maturation.^[^ ^4^ ^]^ Afterward, medium was switched to NMM until day 70 (**Figure** **1**A; Figure S2A, Supporting Information). Autonomous spheroid fusion into large spherical structures was observed from day 10 onwards. The NCs reached a mean diameter of 2100 µm (±115 µm) on day 42 (Figure S2B, Supporting Information). H&E staining showed a uniform outer layer of tangentially aligned cells at the periphery and a core with packed cells (Figure 1B). RT‐qPCR showed an increase in transcripts for the forebrain marker *FOXG1* (Figure S2C, Supporting Information) and presence of dorsal forebrain transcripts *EMX2* and *OTX1* ^[^ ^40^ ^]^ (Figure S2D, Supporting Information). This, together with the decrease in *EMX1* transcripts accompanied by a significant increase over time in ventral forebrain transcripts (*DLX1, DLX5, LHX6, and SHH;* Figure S2E, Supporting Information) suggested that the concentroids have a forebrain identity. However, immunostaining of day 30 and 70 concentroids identifying low or no signal of both dorsal or ventral forebrain markers. FOXG1/TUJ1 positive staining cells were detected initially in the periphery and core region, but by day 70 these were found mainly in the concentroid core (Figure 1C). The core region was also enriched for PAX6^+^ cells (indicative of outer radial glia), which was surrounded by a uniform outer layer of MAP2^+^ mature neurons (Figure 1E) and NESTIN^+^ immature neurons (Figure 1H), resembling the neural plate‐like structure formed in cortical spheroids.^[^ ^4^ ^]^ Both the PAX6^+^ core and MAP2^+^ periphery region increased in size from day 30 to 70 (Figure 1F). The presence of cells staining positive for the post‐mitotic neuron marker *NeuN* in the core region and later in the periphery suggests an inside‐out maturation of neuronal cells in the NC (Figure S2F, Supporting Information). In the periphery, concentric lamination of CTIP2^+^ cells (but few SATB2^+^ cells; data not shown) was detected (Figure 1D). Progressive upregulation of *DLX2*, *GAD1*, and *GAD2* transcripts suggested interneuron development^[^ ^41^ ^]^ (Figure 1G), consistent with the presence of GAD1^+^ cells in the core region (Figure 1H). In addition, co‐expression of the vesicular glutamate transporter *vGLUT1* and the postsynaptic *PSD95* transcripts indicated presence of glutamatergic excitatory neurons (Figure S2G), corroborated by immunolabelled PSD95^+^ cells initially in the core region which extended into the periphery by day 70 (Figure S2I). Although *ISLET1* increased over time at both the transcript and protein level (Figure S2H,J, Supporting Information), two other motor neuron markers, *CHAT* and *HB9*, remained relatively low, suggesting the absence of motor neurons, consistent with ventral forebrain fating.^[^ ^42^ ^]^ Hence, the concentroids were of forebrain identity, whereby the core was enriched with radial glial cells (PAX6^+^) and interneurons, surrounded by a layer of CTIP2^+^ neurons, and a uniform outer layer mixed with immature (NESTIN^+^) neurons and mature glutamatergic neurons (MAP2^+^ and PSD95^+^).

**Figure 2:**
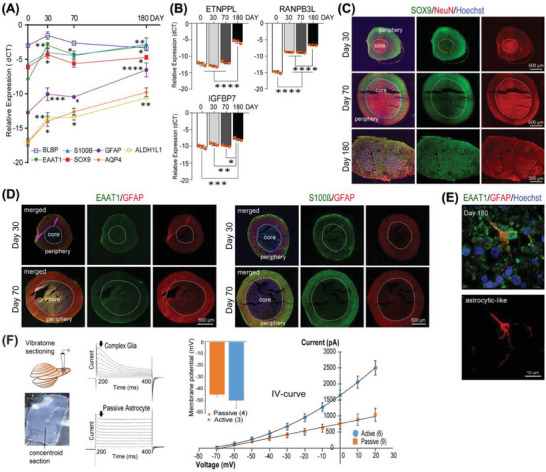
Astrogliogenesis and maturation of NPC within the neural concentroid. A,B) RT‐qPCR analysis for early astroglia (*BLBP*, *EAAT1*, *SOX9*, *S100β*), more astrocyte‐specific (*GFAP*, *AQP4*, and *ALDH1L1*) and mature astrocytes (*ETNPPL, RANPB3L, IGFBP7*) markers over 180 days. (*n* = 3, mean ± SD, duplicate measurements; unpaired *t*‐test compared to day 0 (*p < 0.05, **p < 0.01, ***p < 0.001, ****p < 0.001). C) Immunostaining for SOX9 and NeuN to show astroglia and neurons distribution in the concentroids at each time points. D) Immunostaining for key astrocyte markers GFAP and S100β localized chiefly in the concentroid periphery on days 30 and 70, restricted expression of AQP4 in the core region and limited expression of EAAT1^+^ at the periphery on days 30 and 70. E) Representative 3D reconstructed confocal images showing GFAP^+^ astrocyte‐like cell. F) Electrophysiology measurement of agarose‐embedded concentroid slices on day 180 showing presence of complex glia and passive astrocytes, defined based on distinct membrane potential (mean ± SEM; *n* = 3 active and *n* = 4 passive cells) and electrophysiological properties (mean ± SEM; *n* = 6 active and *n* = 9 passive cells). Nuclei were counterstained with Hoechst.

The astroglial transcripts *SOX9* and *BLBP* were detected early during concentroid culture. By contrast, transcripts for the general astrocyte transcripts (*EAAT1, S100β*, *GFAP, AQP4, ALDH1L1*) (**Figure** **2**A) and mature astrocyte transcripts (*ETNPPL, RANPB3L, IGFBP7* ^[^ ^37^ ^]^; Figure 2B) increased progressively over time, and were highest when cultures were extended until day 180, consistent with what is also seen in cortical organoids.^[^ ^4^, ^43^ ^]^ At earlier time points, SOX9 positive cells were mainly found at the periphery in contrast to *NeuN*‐positive cells in the core region (Figure 2C). However, the SOX9^+^ cells became randomly distributed throughout the construct by day 180. Immunostaining also revealed co‐expression of GFAP, S100β, and EAAT1 in the periphery (Figure 2D,E). Whole‐cell current clamp recordings of cells at the periphery of day 180 concentroid slices identified two astroglial subpopulations (namely complex glia and passive astrocytes) that displayed distinct electrophysiological properties (Figure 2F). The complex glia had an average membrane potential of −49.9 ± 6.48 mV (*n* = 3) and showed electrical stimulation‐induced outward rectifying current (starting around −30 mV), which indicates an active current component response (*n* = 6). The passive astrocytes (n = 9) were characterized by a linear *I–V* relationship and a membrane potential of −44.9 ± 1.75 mV (*n* = 4). Passive electrophysiological properties have been reported to be a signature for mature astrocytes.^[^ ^44^ ^]^ This suggests astroglia maturation into at least two astroglial subpopulations that are reportedly important to shape astrocytic networks during brain development,^[^ ^45^ ^]^ which co‐localized with mature neurons in the concentroid periphery, paralleling temporal cortical development in vivo.^[^ ^46^, ^47^ ^]^

### 2.2 Formation of Pro‐Angiogenic Core in Long‐Term Cultured Neural Concentroids

**Figure 3:**
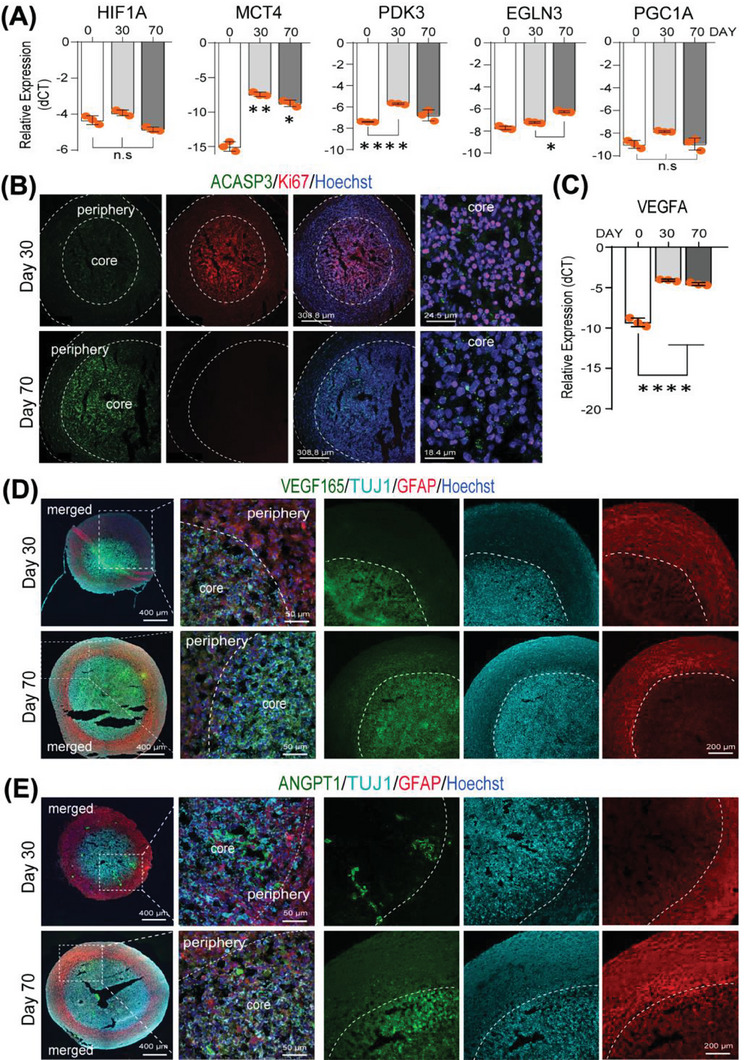
Hypoxia, stress and pro‐angiogenic responses within the neural concentroids. A) Gene expression for key hypoxia markers in the concentroids over 180 days. B) Immunostaining for markers for cell apoptosis (ACASP3) and proliferation (Ki67) on days 30 and 70. C) RT‐qPCR for *VEGF‐A* over 70 days of culture. D,E) Immunostaining for VEGF_165_ and ANGPT1 proteins co‐localized with centrally localized TUJ1^+^ neuronal but not peripheral GFAP^+^ astrocyte marker. All data shown are mean ± SD; *n* = 3, duplicate measurements, one‐way ANOVA with Tukey's multiple comparisons test. Nuclei were counterstained with Hoechst.

Due to the large diameter of NC, we hypothesized that central hypoxia would be present. Indeed, RT‐qPCR demonstrated high *HIF1A* transcript level already on day 0 (i.e., in 2D expanded NPC used to generate spheroids) which persisted until day 70 in the concentroids (**Figure** **3**A). Downstream HIF1A target gene transcripts (*EGLN3, PDK3; except PGC‐1α*), and in particularly the monocarboxylate transporter (*MCT4*) (normally expressed by astrocytes and responsible for lactate production to support the energy demand of neurons^[^ ^48^ ^]^) increased significantly from day 30 onwards. Immunostaining for Ki67 revealed active cell proliferation in the core region on day 30 which decreased afterward (Figure 3B). This was associated with increased signs of cell apoptosis, as activated caspase 3 (aCASP3) was detected in the core region on day 70. Additionally, the *VEGF‐A* transcript levels increased significantly on day 30 and remained high until day 70 (Figure 3C). Immunostaining revealed abundant VEGF_165_ (Figure 3D) and ANGPT1 staining (Figure 3E) in the core region at all time‐points, which co‐localized with the TUJ1 signal but less in the GFAP^+^ periphery. This data suggests that VEGF_165_ and ANGPT1 might be secreted by neuronal, not astrocytic cells. Altogether, these findings indicated the formation of a pro‐angiogenic core in the concentroids, which formed the basis to test if vascularization throughout the concentroid would be possible to improve maturation by co‐culture with iPSC‐derived endothelial cells (EC).

### 2.3 Vascularizing Neural Concentroids Revealed Insights into Neuro‐Astroglial‐Endothelial Interactions

**Figure 4:**
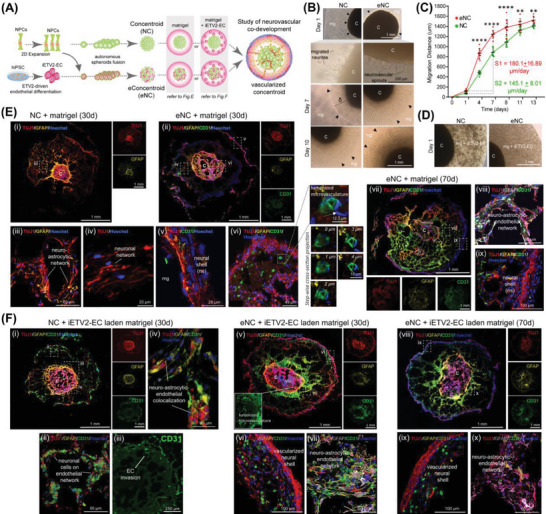
Neuro‐astroglial‐endothelial interactions at the modeled peri‐neural vascular interface. A) Schematic for the generation of neural concentroids (NC) and iETV2‐EC incorporated concentroids (eConcentroids (eNC)) for the study of neurovascular development. B) Representative brightfield images showing out‐migrated neurites (from NC) and neurovascular sprouts (from eNC) into matrigel (*mg*) on days 1, 7, and 10, respectively. The cell migration front is indicated by black arrow heads. The location of the concentroid is labelled as “C”. C) Image‐based quantification of cell migration distance (δ) and speed calculation in matrigel from NC and eNC (S1 = 180.1 ± 16.89 µm day^−1^ versus S2 = 145.1 ± 8.01 µm day^−1^, respectively; mean ± SD, *n* = 3, triplicate measurements; two‐way ANOVA Sidak's multiple comparisons test: **p < 0.01, ****p < 0.0001). D) Representative brightfield images of endothelial network formation by iETV2‐EC in matrigel embedded with NC and eNC on day 1. E) Confocal images showing the cytoarchitecture and spatial distributions of neuronal (TUJ1), astrocytic (GFAP), and EC (CD31) cells within the formed cellular networks (iii, iv, viii), neural shell (ns; v, ix), and in the (i) NC and (ii) eNC that were embedded in matrigel alone for 30 and 70 days (for eNC only; vii). (vi) 3D confocal reconstruction of lumenized microvasculature‐like structures formed in the concentroids at high magnification. White arrow showing the direction of step‐wise cross‐section projection images at 1 µm interval. F) Confocal images showing the cytoarchitecture and spatial distributions of neuronal (TUJ1), astrocytic (GFAP), and EC (CD31) cells within the formed cellular networks (ii, iv, vii, x), neural shell (ns; vi, ix), and in the (i) NC and (v) eNC that were embedded in iETV2‐EC laden matrigel for 30 and 70 days (viii; for eNC only). (iii) Immunostaining showing CD31^+^ signals within the NC, indicates vascularization of the concentroids due to the invasion of iETV2‐EC from the matrigel. Nuclei were counterstained with Hoechst.

Others and we reported recently that activation of the ETV2 transcription factor (TF) in hPSCs creates very fast and homogenous ECs.^[^ ^28^, ^49^, ^50^, ^51^ ^]^ We first assessed PSC‐to EC differentiation efficiency in NMM medium using the doxycycline‐inducible ETV2 overexpression hPSC line (Figure S3, Supporting Information) and validated its EC identity via bulk RNA sequencing (RNAseq) (Figure S4, Supporting Information). We then leveraged the pro‐angiogenic core created in the concentroids to assess the effect of iETV2‐ECs incorporated in/around concentroids on neuronal and astroglial behavior (**Figure** **4**A). We embedded the concentroids, created of NPCs only (NCs) or NPCs together with ECs (eNCs) in matrigel droplets. We observed cell sprouting from the NC and eNC into the matrigel (Figure 4B; *black arrow heads*), with cells migrating till the edge of the matrigel droplets by ± day 13. Measuring the distance of the migration front (δ) revealed a significantly higher cell migration speed from eNCs than from NCs (Figure 4C), demonstrating that presence of EC in eNCs enhanced cell outgrowth.

When NCs not containing ECs were embedded in matrigel droplets, immunostaining on day 30 showed that cells migrated out from the NCs formed extensive cellular networks (Figure 4Ei)) comprised of TUJ1^+^ and GFAP^+^ cells (Figure 4E(iii)). By contrast, when endothelium‐containing concentroids (eNCs) were embedded in matrigel droplets, cells migrating out from the eNC were mainly TUJ1^+^ and to a lesser extent, CD31^+^ cells (Figure 4E(ii,iv)) which then created a ≈25 µm cell thick layer of TUJ1^+^ and some CD31^+^ cells at the edge of matrigel droplets (Figure 4E(v)). At high magnification, 3D confocal reconstruction revealed lumenized endothelial structures in the eNC outer neural shell (Figure 4E(vi)). In contrast to NCs not containing ECs, the majority of GFAP^+^ cells did not migrate from the eNC into the surrounding matrigel on day 30. By day 70 (Figure 4E(vii)), a more extensive cellular network could be observed in the matrigel surrounding eNCs (Figure 4E(viii)), with a thicker (≈100 µm thick) peripheral neuron containing shell at the periphery of the matrigel droplet (Figure 4E(ix)). This was accompanied by a decrease in size of the central eNC. Moreover, at this later timepoint, GFAP^+^ cells (indicated by asterisks) could also be found in the TUJ1^+^/CD31^+^ networks in the matrigel and outer neural shell. Thus, when ECs are present, ECs but not astroglial cells support the initial neuronal cell outgrowth. Moreover, when ECs are present, the out‐migrated neural cells created a new outer shell of neural and vascular cells initially without astroglial cells, but at later times also astroglial cells.

In a second set‐up, we incorporated ECs in the matrigel droplet wherein either NCs or eNCs were embedded. The ECs present in the matrigel droplet quickly formed an extensive network (Figure 4D). When NCs were cultured in EC‐laden matrigel droplets, immunostaining on day 30 identified again mainly TUJ1^+^ cells that migrated out from the NC and co‐localized with the CD31^+^ endothelial network formed in the matrigel (Figure 4F(i,ii)). In addition, some CD31^+^ cells were detected in the NC indicating EC invasion (Figure 4F(iii)). GFAP^+^ cells remained predominantly in the NCs, and GFAP^+^/TUJ1^+^/CD31^+^ networks were formed at the border of the NCs (Figure 4F(iv)). When eNCs were embedded in EC‐laden matrigel, large numbers of both TUJ1^+^ and GFAP^+^ cells migrated from the eNC into the matrigel on day 30 (Figure 4F(v)), again also co‐localizing with CD31^+^ endothelial networks formed in the matrigel (Figure 4F(vii)). A prominent TUJ1^+^/GFAP^+^/CD31^+^ neural shell (≈150 µm thick) was formed at the edge of the matrigel droplet (Figure 4F(vi)) while lumenized endothelium could now also be detected inside the eNC (Figure 4F(v) inset). On day 70 (*only for eNC;* Figure 4F(viii)), the neural shell (Figure 4F(ix)) and TUJ1^+^/GFAP^+^/CD31^+^ cellular networks (Figure 4F(x)) could still be observed. These findings further demonstrate an important role for ECs in governing neuronal and astroglial cell out‐migration. Our data are in line with other studies demonstrating that migration of neuronal cells can be guided either by astrocytic processes^[^ ^52^ ^]^ or by blood vessels^[^ ^53^ ^]^ during cortex and vasculature co‐development in the brain.

### 2.4 Premature Exposure to Endothelial Cells also Affected Ventricular Zone Formation in Cerebral Organoids

**Figure 5:**
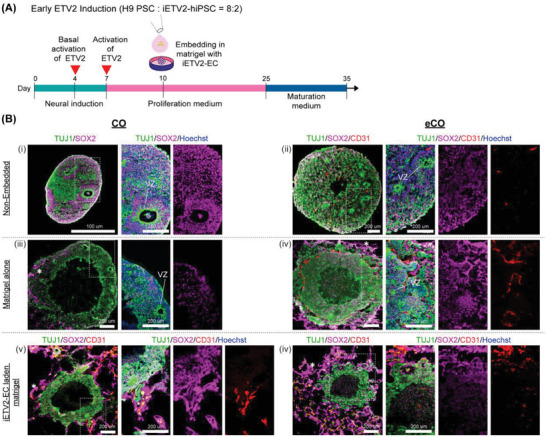
Effect of matrigel embedding and/or the presence of iETV2‐EC on the radial organization of SOX2^+^ cells in the ventricular zone (VZ) in cerebral organoids. A) Schematic of the culture protocol to study the effect of ETV2 activation time points and matrigel embedding on cerebral organoid development. B) Immunostaining showing: (i) preserved and apparent organization of SOX2^+^ cells in the ventricular zone (VZ) of PSC‐derived cerebral organoids (CO); (ii) which was slightly disrupted in PSC/iETV2‐hiPSC (8:2 ratio) derived cerebral organoids (vCO); (iii & iv) largely disrupted when CO and vCO were embedded in matrigel as SOX2^+^ cells were mobilized into the matrigel; and (v & vi) abrogated when CO and vCO were embedded in iETV2‐EC laden matrigel as SOX2^+^ cells were largely mobilized into the matrigel. Nuclei were counterstained with Hoechst. Scale bar = 200 µm.

We next assessed if iETV2‐ECs could also be used to vascularize cerebral organoids (CO), and if presence of ECs would affect the localization of the different neural compartments. We thus generated PSC‐derived COs and vascularized COs (eCOs; by allowing organoid formation from a mix of iETV2 and wild type PSCs; PSC:iETV2‐hiPSC ratio = 8:2). To generate iETV2‐ECs in situ, we activated the *ETV2* transgene by addition on day 4 and 7, respectively, low and high concentrations of doxycycline for basal and full transgene activation (**Figure** **5**A). Immunostaining of DIV35 eCO revealed CD31^+^ vascular structures and relatively smaller and scattered ventricular zones (VZ) (Figure 5B(ii)) in which the radial organization of SOX2^+^ cells (but not TUJ1^+^ cells) was less apparent than that of CO not containing ECs (Figure 5B(i)). We also embedded CO and eCO in matrigel alone or in iETV2‐ECs laden matrigel. In matrigel alone, we observed VZ‐like structures in the CO, however the SOX2^+^ cells were mostly found outside the VZ (indicated by asterisk; Figure 5B(iii)). In eCO, the radial organization of SOX2^+^ in VZ was more disrupted (Figure 5B(iv)), whereby the SOX2^+^ and TUJ1^+^ cells appeared to spread into the matrigel, and CD31^+^ cells were found mainly at the periphery. In iETV2‐EC

laden matrigel, the VZ formation was abrogated in both CO and eCO, whereby SOX2^+^ and TUJ1^+^ cells spread massively into the matrigel (Figure 5B(v,vi)). These findings suggest that even if matrigel alone can affect VZ formation by promoting SOX2^+^ cell mobility away from the VZ, incorporation of iETV2‐EC in the cerebral organoid and even more so in the surrounding matrigel abrogated VZ formation due to out‐migration of SOX2^+^ cells.

### 2.5 PDGFRβ^+^ Cells were of NPC‐Origin and Highly Co‐Localized with CD13 Positivity but not αSMA Suggesting Brain Capillary Pericyte‐Like Cell Identity

**Figure 6:**
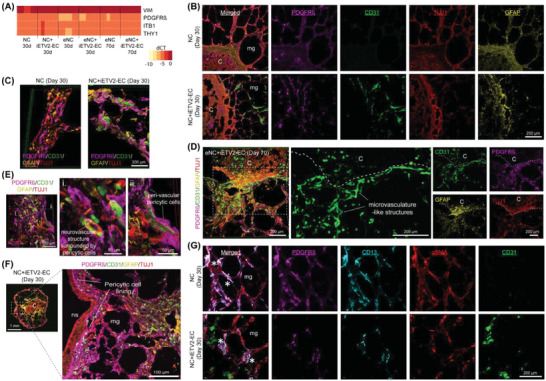
Pericytic‐like cells present in concentroids are derived from the NPCs not mesodermal ECs. A) RT‐qPCR analysis of pericyte markers VIM, PDGFRβ, ITB1 and THY1 expression in NC and eNC. Data shown are mean ± SD (*n* = 3, duplicate measurements). B) Immunostaining demonstrates that PDGFRβ^+^ cells are present in the engineered constructs regardless of the presence of iETV2‐EC in matrigel (*mg*). C) 3D confocal reconstructed images for PDGFRβ^+^ cells, TUJ1^+^ neuronal and GFAP^+^ astrocytic cells, and CD31^+^ ECs. D) The PDGFRβ^+^ cells were found mainly adjacent to the 3D microvasculature‐like structures formed at the interface (dotted lines) between eNC and iETV2‐laden matrigel on day 70. E) Representative 3D confocal reconstructed images showing lumenized vessel structure and elongated microvasculature surrounded by PDGFRβ^+^ cells. F) PDGFRβ^+^ cells lining the interface of TUJ1^+^ outer layer at neural shell (ns) wherein eNC was embedded in iETV2‐EC laden matrigel for 70 days. G) Immunostaining revealed co‐localization of PDGFRβ and CD13 labeling (*) but not αSMA.

As RT‐qPCR revealed high transcript levels of putative pericyte markers (*VIM, PDGFRβ, ITB1, THY1*)^[^ ^54^ ^]^ in both matrigel embedded NCs and eNCs (**Figure** **6**A) and we did not add a population of pericytes, we assessed if pericytes were generated from the NPC or EC population. Immunostaining demonstrated that PDGFRβ^+^ cells were present in all cultures, regardless of the presence of iETV2‐ECs, indicating that PDGFRβ^+^ cells were of NPC origin (Figure 6B,C). At the interface between the concentroid and matrigel, PDGFRβ^+^ cells were co‐localized with TUJ1^+^/GFAP^+^/CD31^+^ networks (Figure 6D). At high magnification, 3D confocal reconstruction revealed peri‐vascular localization of PDGFRβ^+^ cells (Figure 6E) as well as in the neural shell (Figure 6F). Human brain pericytes express PDGFRβ, αSMA, CD13, NG2, CD146, and desmin, while vascular smooth muscle cells express higher levels of αSMA, CD146, and desmin than pericytes.^[^ ^55^ ^]^ We demonstrated that PDGFRβ^+^ cells stained positive for CD13 but to a much lesser extent αSMA (Figure 6G), suggesting brain capillary pericyte‐like cell but not smooth muscle‐like cell formation from NPCs.^[^ ^56^ ^]^

### 2.6 Vascularizing Neural Concentroids Enhance Transcriptional Dynamics Associated to Neurogliogenesis

**Figure 7:**
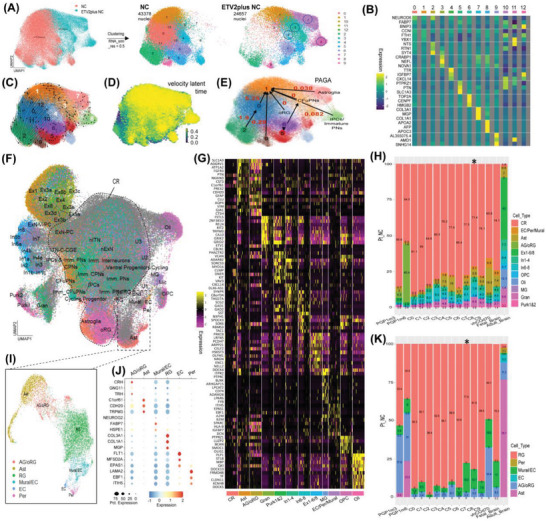
Single nucleus transcriptomic analysis of neural cell subtypes maturation and diversity. A) Uniform Manifold Approximation and Projection (UMAP) plots of merged Seurat objects for ETV2plus NCs and NCs and the 13 clusters obtained at resolution of 0.5. Cluster 3, 4, 7, and 8 (indicated by circles) were differentially enriched in ETV2plus NC as compared to NC. B) Heatmap showing the expression of the identified top‐3 gene markers for each cluster across the 13 clusters obtained from the merged Seurat objects. C,D,E) Dynamic modeling of RNA velocity stream, velocity latent time, and the partition‐based graph abstractions (PAGA) velocity pseudotime mapping of each cluster identified from the merged Seurat objects. F) UMAP plot showing the original cell identities from the reference Seurat objects from brain organoids (aged 3 (PGP1m3) and 6 months (PGP1m6)), vascularized human brain organoids (vhCOd70; day 70), fetal and adult human brains. CR = core region; Mic/MG = microglia; OPC = oligoprogenitor cells; ln = Inhibitory neurons; Gran/Purk1&2; granular cells and purkinje cells 1 & 2; PNs = projection neurons; CPNs = callosal projection neurons; IPCs = intermediate progenitor cells; CFuPNs = corticofugal projection neurons; EC = endothelial cells; Per = pericytes; Mural = mural cells; Ast = astrocytes; oRG = outer radial glia; RG = radial glia; Imm. = immature cells; nITN = newborn interneuron; Ex = excitatory neurons; nExN = newborn ExN; ExN‐PC = excitatory neuron – progenitor cells; ExN‐V‐PC = excitatory neuron‐ventral‐progenitor cells; U1, U2 & U3 = Unknown cell type 1, 2 and 3. G) Heatmap showing top‐10 gene markers for each cell type identified from (F). H) Cell‐type composition analysis of each cluster identified from the Seurat objects of ETV2plus NCs and NCs after integrated with the reference Seurat objects. *y*‐axis = percentage of nuclei counted (Pt_NC). I,J,K) UMAP plot for the subset cluster 22 (EC/Per/Mural) and astrocytes‐associated clusters, and the dotplot showing the top‐3 cell‐type specific gene markers, and the cell‐type composition analysis for the integrated Seurat objects. Cluster 7 and 8 showed cell‐type composition reminiscent fetal human brain as compared to adult human brain and brain organoids (marked by asterisk).

Single nucleus RNA sequencing (snRNAseq) analysis was performed to study the effect of vascularizing NCs, by comparing eNCs embedded in EC‐laden matrigel (ETV2plus NC, day 30; 24 657 nuclei) with NCs embedded in matrigel without ECs (NC, day 30; 43 378 nuclei). Functional enrichment analysis of the top‐20 highly variable genes for both conditions (Table S1) showed that ETV2plus NCs were enriched with pathways associated to nervous system development, whereas NCs were enriched with necrotic cell death‐ and hypoxia‐related pathways (Figure S5A, Supporting Information). The Uniform Manifold Approximation and Projection (UMAP) of the merged Seurat object (**Figure** **7**A; resolution RNA_snn = 0.5) revealed four cell clusters (i.e., cluster C3, C4, C7 and C8 out of 13 clusters; indicated by circles) that were differentially enriched in ETV2plus NCs (see Figure 7B; Table S2 for their top‐3 gene markers). By referencing to the online interactive single‐cell portal of human brain organoid databases (schBO)^[^ ^57^ ^]^ (https://singlecell.broadinstitute.org), we putatively denoted C3 as corticofugal projection neurons (CFuPNs), C4 as outer radial glia (oRG), C7 as astroglia (AG), and C8 as intermediate progenitor cells/immature projection neurons (IPCs/Imm. PNs).

RNA velocity analysis with high confidence (indicated by the high coherence of velocity vector field of >0.995, Figure S5C, Supporting Information) allowed us to gain insight into the transcriptional dynamics. The dynamic model^[^ ^58^ ^]^ showed that the RNA velocity stream for each cluster was mainly delineated towards the C1 cluster (Figure 7C). Based on the schBO databases, the C1 cluster was a mixed cell pool of developing intermediate neuronal and glial cells, including outer radial glia (oRG), astroglia (AG), intermediate progenitor cells (IPCs), ventral precursors (VP), immature interneuron (imm. ITN), immature and mature corticofugal (CFuPNs) and callosal projection neurons (CPNs) (Figure S5E, Supporting Information). Additionally, the relatively higher RNA velocity latent time in C1, C3, C4, C7, and C8 suggested that ETV2plus NCs produced more cells that were in later transcriptional stages as compared to NCs (Figure 7D). Moreover, the partition‐based graph abstraction (PAGA) velocity pseudotime mapping showed that these four clusters were transited on average ∼83 times faster to C1 than the other five cell clusters enriched in NCs (i.e., C0, C2, C5, C6, C9) (Figure 7E; Table S3). This is corroborated by the inferred high RNA velocities for most of the top marker genes of these four clusters (Figure S5B, Supporting Information; Table S4), which confirmed transcriptional upregulation of these cluster‐specific marker genes. In fact, *NEFL, TTR, DCN* and *APOA2* were ranked the most dynamic velocity genes for clusters C3, C4, C7, and C8, respectively (Table S5). The estimated high velocity length for C4 (Figure S5D, Supporting Information) indicated that C4 had the highest rate of transcriptional dynamics, implying highest differentiation rate. Thus, the presence of ECs enhanced transcriptional activity of putative driver genes specific to the clusters C3 (CFuPNs), C4 (oRG), C7 (AG), and C8 (IPCs/Imm. PNs), which were differentially enriched in ETV2plus NCs as compared to NCs.

### 2.7 Vascularizing Neural Concentroids Enhances Neural Cell Diversity Reminiscent of Fetal‐To‐Early Mouse and Early Fetal Human Brain Development

To further annotate the cell diversity in ETV2plus NCs and NCs, we integrated our snRNAseq datasets with the published sc/snRNAseq datasets of human brain organoids (month 3 and 6),^[^ ^57^ ^]^ vascularized human brain organoids (vhCOd70; day 70),^[^ ^28^ ^]^ fetal human brain cortex (∼6 – 37 post‐conception week (pcw))^[^ ^59^ ^]^ and adult human brain cortex (51‐year‐old female).^[^ ^60^ ^]^ The integrated UMAP (Figure 7F; Figure S5F, Supporting Information) showed most cells derived from ETV2plus NCs and NCs, vhCOd70, fetal human brain cortex, and brain organoids could be found in a big core cluster (CR) (∼ 54.5% to 89%, Figure 7H; expectedly low in adult brain, 1.9% only), which consisted of a mixture of newborn neuronal cells, intermediate progenitors, mature neuronal cells and radial glia (RG) that could not be segregated into individual cell clusters even at high resolution (i.e., integrated RNA_snn_3.5). Surrounding this core cluster, 10 smaller clusters of cells annotated as oligodendrocytes (Oli), microglia (Mic/MG), oligodendrocyte‐progenitor cells (OPC), astroglia (AG) and outer RG (oRG), astrocytes (Ast), granular and purkinje cells (Gran/Purk1&2), inhibitory neurons (In), and excitatory neurons (Ex). Endothelial cells (EC/End), mural cells (Mural) and pericytes (Per) co‐clustered together as cluster 22. The gene markers specific to each cell cluster are shown in Figure 7G. Quantitatively, cluster C8 contained the highest portion of these cell types (≈28.5%) and was most similar to vhCOd70 (34.2%) and fetal human brain (25.7%) (Figure 7H). The percentages of these cell types in C3, C4, and C7 were in general also higher than in clusters derived from NCs. In contrast, brain organoids at month 3 (i.e., PGP1m3) contained only 13.5% of these cell types, which increased to 44.7% at month 6, due to a nearly 10‐fold increase in AG/oRG compartment. Subsequently, we subsetted cluster 22 and the Ast‐associated clusters (i.e., cluster 10, 23, 30, and 31), and thus further segregated and annotated the Ast, AG/oRG, RG, Mural/EC, EC, and Per clusters (Figure 7I) which feature individual cell type‐specific marker genes (Figure 7J). Quantitatively, the Mural/EC and AG/oRG composition of cluster C7 and C8 resembled that of fetal human brain to a lesser extent vhCOd70 in term of AG/oRG percentage (Figure 7K). By contrast, brain organoids were more enriched with AG/oRG and Ast populations and with much lower RG percentage. The adult brain was characterized by high Ast (76.7%) and low RG (2.8%) populations, containing 3.2% Per, and 5.3% EC (i.e., true brain EC) which was not present in ETV2plus NCs and NCs, nor in fetal human brain sample, and a negligible amount in brain organoids (0.2%–0.5%; somewhat unexpected as EC is of mesodermal not ectodermal origin). This demonstrated that eNC and vhCO did not contain cells with typical transcriptional features for mature adult brain ECs and pericytes, but that the Mural/EC clusters appears to represent a developing cell population with intermediate identity between mural and ECs. Hence, the cells staining positive for pericyte markers, described in Figure 6 may not represent mature pericytes.

Furthermore, by projecting the expression level of marker genes in ETV2plus NCs and NCs to that of the developing mouse databases spatially via VoxHunt^[^ ^61^ ^]^ analysis, we found that ETV2plus NCs and NCs were correlated more to mouse forebrain and hindbrain than midbrain of E18.5 and postnatal P4 and P14 (Figure S6A,B, Supporting Information: *cross‐section views;* Figure S6C, Supporting Information: *3D models*). After data deconvolution, we estimated ∼60 to 80% forebrain (i.e., pallium, subpallium, diencephalon, and hypothalamus), ∼20 to 40% hindbrain (i.e., prepontine hindbrain, pontine hindbrain, and medullary hindbrain), and ≈2 to 4% midbrain phenotypes (Figure S6D, Supporting Information), which were comparable between ETV2plus NCs and NCs. Mapping to the human BrainSpan database based on the top‐10 regional markers extracted from ISH mouse brain atlas (at E18.5, P4, and P14 stages), we computed consistently higher similarity of ETV2plus NCs and NCs to the neo‐cortex (NCx) and ganglionic eminences (GE) than other structures of developing fetal human brain at 8–9 pcw (Figure S6E, Supporting Information). Collectively, these findings further corroborated our earlier notion that the generated NCs possessed cell‐type compositions reminiscent of fetal human brain, even if minute differences were found between ETV2plus NCs and NCs. Within the stipulated experimental time frame in this study, high correlation of the generated NCs to the late prenatal and early postnatal mouse brain on one hand, and early postconception human brain development on the other hand, is in line with the fact that cortical development in mouse brain organoids is reportedly very much faster than in human brain organoids.^[^ ^62^ ^]^

### 2.8 Gene Regulatory Networks in Vascularized Neural Concentroids are Linked to Anterior/Posterior Pattern Specification in General, and Vasculature Development, Extracellular Matrix Constitution, and/or Glial Cell Proliferation, in Particular

**Figure 8:**
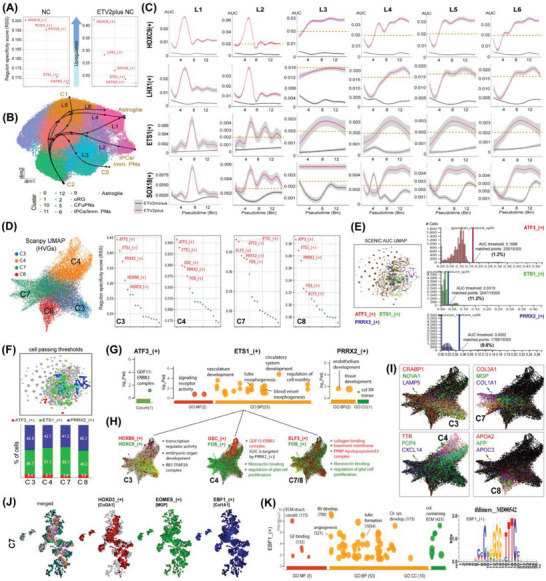
Inferring cluster‐specific gene regulatory network activation and functional pathway enrichment in vascularized neural concentroids. A) Differentially regulated top‐5 regulons in ETV2plus NCs as compared to NCs. B) The inferred six lineage trajectories by *Slingshot* analysis lead to different end clusters (i.e., C1, Astroglia, IPCs/Imm. PNs, and C5), whereby C2 was denoted as the initial cell cluster/progenitor cells. C) Plots of regulon activity levels of *HOXC9_*(+), *LHX1_*(+), *SOX18_*(+) and *ETS1_*(+) along the pseudotime (bin = 15) for the identified six lineage trajectories in ETV2plus NCs and NCs. D) The Scanpy UMAP plot based on the highly variable genes (HVGs), and the predicted top‐5 regulons specific to C3, C4, C7 and C8 (ranked based on the regulon specificity score (RSS)) via pySCENIC analysis. E) The SCENIC_AUC_UMAP plot showing the distribution of cells with either of the thresholds and the percentage of cells passing the thresholds. F) The SCENIC_AUC_UMAP plot showing the distribution of cells passing the regulon thresholds, and the estimated percentage of cells active in the respective regulons in C3, C4, C7 and C8. G,H) Functional enrichment analysis of the predicted top‐3 common regulons and two lower‐scored regulons specific to each cluster via g:Profiler. I) The Scanpy UMAP plots showing cluster‐specific expression of the identified gene markers for C3, C4, c7, and C8 via snRNAseq analysis. J,K) The SCENIC_AUC_UMAP plots showing co‐expression of regulons targeting to gene markers identified for C7 via snRNAseq analysis, and the functional enrichment analysis of *EBF1_*(+) regulon via g:Profiler. Col XII trimer = collagen XII trimer; ECM struct. constit. = extracellular matrix structural constituent; GF binding = growth factor binding; BV develop. = blood vessel development; cir. sys. develop. = circulatory system development; col. containing ECM = collagen containing ECM.

We also performed Single‐Cell Regulatory Network Inference and Clustering (pySCENIC^[^ ^63^ ^]^) analysis to predict cluster‐specific TFs and gene regulatory network (GRNs) activity that might be responsible for driving neural cell maturation and diversity reminiscent of fetal human brain observed in ETV2plus NC as compared to NC in general, and in C7 and C8 as compared to C3 and C4 in specific. By inferring the adjaciencies of GRNs based on co‐expression patterns, we identified TFs and their target genes (Table S6). In general, the analysis revealed three main regulons [i.e., *HOXC9_*(+), *LHX1_*(+), and *SOX18_*(+)] that were upregulated in ETV2plus NC as compared to NC (**Figure** **8**A; Table S7). Functional enrichment analysis showed that the regulons *HOXC9_*(+) and *LHX1_*(+) are linked to the enrichment of embryonic development pathways including anterior/posterior pattern specification (P_adj_ = 9.905 × 10^−9^ to 1.342 × 10^−15^) and regionalization (P_adj_ = 1.861 × 10^−7^), while the regulon *SOX18_*(+) is associated with blood vessel development (P_adj_ = 5.610 × 10^−10^) or angiogenesis (P_adj_ = 2.212 × 10^−7^) (Figure S7A–C, Supporting Information). Based on the RNA velocity analysis, we inferred the possible lineage trajectory by performing *Slingshot* ^[^ ^64^, ^65^ ^]^ analysis with C2 denoted as the initial cell cluster (i.e., putative progenitor cells; top‐4 marker genes = *MALAT1*, *SOX4*, *STMN1* and *STMN2*; Table S8). The analysis revealed six lineage trajectories (Figure 8B; Figure S7D, Supporting Information), which individually led to C7 (Astroglia; via L1, L4, and L6), C8 (IPCs/Imm. PNs; via L2), C5 (via L3), and C1 (via L5) as end points, respectively. Plotting the regulon activities (including *ETS1_*(+)) in pseudotime (bin = 15) once again confirmed higher activity levels of the four regulons in ETV2plus NC compared to NC (Figure 8C). Lineage‐wise, L1 differed from L4 and L6 as its regulon activities were transient (peaks around bin4 or bin8), while expression was higher (indicated by dashed yellow lines) and prolonged in L4 and L6. The regulon activities in L5 were comparable to L4 and L6. L2 exhibited higher and prolonged *ETS1_*(+) and *SOX18_*(+) activities than L1, whereas L3 was characterized by a constant elevated *SOX18_*(+) activity. We found that within each regulon, *HOXC9_*(+) and *LHX1_*(+) are inter‐targeted, whereas *ETS1_*(+) is targeted by *SOX18_*(+).

The cluster C3 (CFuPNs), C4 (oRG), C7 (AG) and C8 (IPCs/Imm. PNs) shared three common top‐ranked regulons [i.e., *ATF3_*(+), *ETS1_*(+) and *PRRX2_*(+)], and two other lower‐scoring regulons seemingly specific to C3 [*HOXB9_*(+) and *HOXC9_*(+)], C4 [*GSC_*(+) and *FOS_*(+)], and C7/C8 [*ELF_*(+) and *FOS_*(+)] (Table S9). Based on the Area Under Curve (AUC) values of each regulon, the estimated percentage of cells that passing the AUC threshold was 1.2% for *ATF3_*(+), 11.2% for *ETS1_*(+), and 9.8% for *PRRX2_*(+) (Figure 8E), indicating the percentage of cells that were active in either of these three regulons was low, yet the number of cells active in *ETS1_*(+) or *PRRX2_*(+) was ∼10 times higher than those active in *ATF3_*(+). In fact, C3, C4, C7 and C8 consisted of ∼41 – 55% cells that were active in either *ETS1_*(+) or *PRRX2_*(+) as compared to only ∼3.6 – 6.4% for *ATF3_*(+) (Figure 8F; Table S10), suggesting that *ETS1_*(+) and *PRRX2_*(+) regulons were the key GRN determinants in these clusters compared to *ATF3_*(+). Functional enrichment analysis revealed, among others, an enrichment in GDF15‐ERBB2 complex pathway linked to *ATF3_*(+), vasculature development/signaling receptor activity/cell motility regulation pathways linked to *ETS1_*(+), and endothelium development/col XII trimer pathways linked to *PRRX2_*(+) (Figure 8G). Via iRegulon^[^ ^66^ ^]^ analysis, we mapped the predicted regulatory network of TFs and their co‐expressed target genes for these top‐3 regulons (Figure S7E–G), and thus identified the most enriched TF for regulon *ATF3_*(+) (i.e., *TFAP2A; normalized enrichment score* (*NES*) = *12.69*), *ETS1_*(+) (i.e., *BCL6B; NES = 6.438*), and *PRRX2_*(+) (i.e., *SOX21; NES = 6.262*). Based on the two lower‐scoring regulons, C3 was also enriched with pathways associated to transcription regulator activity, embryonic organ development, and RB1‐TFAP2A complex; C4 enriched with GDF15‐ERBB2 complex, fibronectin binding, and regulation of glial cell proliferation pathways; and C7 and C8 enriched with collagen binding, basement membrane, PRNP‐ApolopoproteinE3 complex, in addition to fibronectin binding and regulation of glial cell proliferation pathways (Figure 8H). We subsequently mapped the cellular activity of the top‐3 gene markers identified in C3, C4, C7, and C8 via snRNAseq analysis above, which again showed cluster‐specific expression of these marker genes (Figure 8I). Moreover, plotting a SCENIC AUC UMAP for C7 alone we demonstrated the co‐expression of novel regulons *HOXD3_*(+), *EOMES_*(+), and *EBF1_*(+), which are known to target the top‐3 marker genes of C7 (i.e., *Col3A1*, *MGP* and *Col1A1*, respectively; Figure 8J). These marker genes coincided with the identified marker genes of AG, as shown in Figure 7J. Of interest, *EBF1_*(+) was found to be a regulon enriched in extracellular matrix (e.g., collagen) structure constitution, growth factor binding, and blood vessel development including angiogenesis and tube formation (Figure 8K). No regulon could be predicted for C8 when a SCENIC AUC UMAP was plotted separately. Altogether, these findings further justified that vascularizing NC‐activated GRNs that may enhance neurovascular development.

## 3 Discussion and Conclusion

In this study, we used human PSC‐NPC‐derived concentroids to study the effect of vascularization on neural cell behavior and development. This model is characterized by several key observations: 1) Fusion of PSC‐NPC spheroids rather than generating brain organoids using methods derived from the seminal study described by Lancaster et al., creates homogenous neural concentroids (NCs). The NCs consisted of a pro‐angiogenic core containing radial glia, glutamatergic neurons, and interneurons, which was surrounded by deep layer CTIP2^+^ neurons and at later time points an outer layer of neurons, and phenotypically and functionally mature astrocytic cells, resembling the outer neural plate of cortical spheroids in a concentric manner; 2) Embedding NCs in matrigel affected cellular organization of the ventricular zone due to out‐migration of neuronal and astroglial cells. This effect was not unique to NCs as it was also observed in COs, thus suggesting a simpler and homogenous neural model that exhibited similar cellular behavior is also a useful alternative brain model as compared to COs with complex yet inhomogeneous cytoarchitecture; 3) When ECs were present, ECs but not astroglial cells were the most important guide for out‐migration of neuronal cells; 4) The NPCs used to create the concentroids spontaneously gave rise to mural/pericyte‐like cells, mimicking the ectodermal^[^ ^67^, ^68^ ^]^ not mesodermal^[^ ^69^, ^70^, ^71^, ^72^ ^]^ origin of cortical pericytes. 5) The presence of ECs significantly affected the cellular make‐up of concentroids, including neuron and astroglia localization and this in a location‐dependent manner; 6) In addition, presence of ECs: (a) hastened neural differentiation; and (b) the cell type composition of eNC resembled fetal human brain more closely than classical COs.

Undoubtedly, the observation that the location of ECs significantly affected neuronal and astroglial out‐migration is intriguing and summons new attention from scientists when aiming at designing an effective vascularization approach. Interestingly, a thick neural shell at the periphery of the matrigel was formed only when ECs were present, highlighting the potential of this co‐culture system to gain insights into the mechanisms by which ECs induce neural migration^[^ ^73^ ^]^ for instance, to improve brain injury repair following stroke, or neurovascular malformations.^[^ ^74^ ^]^ Indeed, we provided evidence that incorporation of ECs in NCs is associated with increased expression of GRNs not only linked to blood vessel development (*SOX18_*(+) and *ETS1_*(+)), but also linked to enhanced embryonal anterior/posterior pattern specification (i.e., *HOXC9_*(+) and *LHX1_*(+)), cell motility/migration (i.e., *EST1*_(+)), and/or ECM formation (i.e., *PRRX2*_(+), *EBF1*_(+), *FOS*_(+) and *ELF3*_(+)). The presence of ECs also hastened neurogenesis from hNPCs (as shown by transcriptional profiling and in line with previous studies^[^ ^75^, ^76^ ^]^). Even if the precise signals underlying this hastened differentiation remain to be elucidated, the identification of cell clusters significantly more abundant in ETV2plus NCs has provided initial insights into GRNs that drive neural maturation and cell‐type diversity in the context of vascularization. Specifically, the TFs/regulons driving hastened neural development were *HOXC9*, *LHX1*, *ATF3*, *ETS1*, and *HOXB9*; and for vascularization *SOX18*, *ETS1*, *PRRX2* and *EBF1*, respectively. These findings could be translated into novel genome engineering strategies to facilitate robust brain mimic generation through augmentation of these GRNs, or for disease modeling by means of gene network perturbations.

Our study also demonstrated that early induction (i.e., day 4) of an EC fate in the COs and even more so in the matrigel surrounding the COs significantly affected ventricular zone (VZ) formation, with an apparent mobilization of SOX2^+^ cells away from the VZ.^[^ ^77^ ^]^ The interaction of SOX2^+^ cells with laminin (either derived from ECs or present in the matrigel) via α6β1 integrin may be the cause of mobilization, which was reported to be crucial to sustain SOX2^+^ stem/progenitor cells proliferation and stemness in the VZ.^[^ ^77^ ^]^ Nevertheless, the nature of EC‐derived cues underlying SOX2^+^ cells mobilization remains elusive.^[^ ^27^ ^]^ More investigations are needed to fine‐tune the optimal time of EC introduction to obtain robust vascularization of brain organoid without jeopardizing cortical neurogenesis.

Based on our snRNAseq analysis, the iETV2‐ECs had an intermediate mural/EC identity compared to ECs found in the adult human brain. This would be in line with a recent study by Couch et al., in which they concluded that even human mural and endothelial cells from the third trimester as well as perinatal and early postnatal stage brain do not yet feature definitive BMEC or pericyte transcriptomes, morphology or subtype classifications.^[^ ^78^ ^]^ Thus, prolonging the culture time might be beneficial to promote further maturation of the mural/EC cells within the NCs with higher brain‐specificity. Additionally, it would be of interest to determine if ECs incorporated in other brain‐region‐specific NCs are fated to a different endothelial phenotype in due time, as there is considerable evidence for transcriptionally heterogeneity^[^ ^53^, ^79^, ^80^, ^81^ ^]^ of ECs present in different regions of the central nervous system (CNS).

In general, recreation of the BBB from PSCs is performed by co‐culture of neural cells, ECs but also mesodermal pericyte progenitors or pericytes.^[^ ^69^, ^70^, ^71^, ^72^ ^]^ Notably, *ETV2*‐driven PSC‐progeny has also been reported to contain cells with pericyte markers, such as PDGFRβ and THY1.^[^ ^28^ ^]^ This suggests that pericytes could be derived from different sources. However, in vivo, cortical pericytes are derived from the neural crest,^[^ ^67^, ^68^ ^]^ not mesoderm. In line with this notion, we demonstrated spontaneous pericyte‐like differentiation from NPCs in concentroids, in the absence of *ETV2*‐PSC‐derived cells. Many of these pericyte‐like cells could be found, as expected, surrounding lumenized vascular structures, and surrounded by astrocytic cells as well as neurons, a configuration one would expect in BBB models. Nevertheless, snRNAseq analysis revealed that these pericyte‐like cells did not attain the same identity as pericytes found in the adult brain (featuring high expression level of *LAMA2*, *EBF1*, and *ITIH5*), suggesting immaturity. In fact, *EBF1* was reported to play an important role in pericytic commitment, including in human brain vascular pericytes.^[^ ^82^ ^]^ Hence, this NC model may facilitate the study of the molecular development for pericyte maturation, the role of pericytes in neuro‐vascular development in the CNS as well as potentially serve as a model for the BBB, for instance, to study the role of pericytes in neuropathology.^[^ ^83^ ^]^ Although iETV2‐ECs and NPCs had built‐in tdTomato and BFP reporter, respectively, these fluorescence reporters were lost after immunostaining thus preventing us from quantifying the number of PDGFRβ^+^ cells in the concentroids in relation to iETV2‐ECs and NPC origin. In future studies, it will be of interest to perform such lineage tracing to determine the contribution of iETV2‐ECs and NPCs to the PDGFRβ^+^ cells, as such to gain further insights into the origin of pericytes within this cell culture system.

Taken together, our approach creates a vascularized 3D human brain culture model consisting of an inner core of neurons, connected by thick bundles of neurites, astroglial, pericytic, and ECs to a highly vascularized outer shell of neurons and astroglia. This model holds potential for studying the development of different neural and vascular cells in a cortex‐mimicking context, the influence of the vasculature on neural cell migration, as well as neo‐neural tissue regeneration in brain injury such as stroke. Some limitations of our study include: (1) The lack of mechanistic understanding on the fusion of PSC‐NPC spheroids and no controllability on the formation of pro‐angiogenic core; (2) Although the concentroids have a much more homogenic cytoarchitecture, the use of predifferentiated PSC‐NPC to create the concentroids prevents study of a number of aspects of embryonic brain development; (3) In the current study we only used a single PSC lines (hESC H9). It will therefore be important to validate the results in additional hPSC lines to demonstrate the robustness of the approach; (4) The electrophysiology study was performed on concentroids but not vascular concentroids, even though it will be of interest to determine the influence of the vasculature of the electrophysiological properties of astrocytes (and neurons). Similar assessment should be performed on vascularized concentroids; (5) Due to the random formation of a non‐perfusable vascular network within and surrounding the concentroids, functional studies of neurovascular interface with physiological relevance were not feasible. Future studies should thus be aimed at, and not limited to, gaining insights into the precise mechanisms of the cellular crosstalk between the neural and vascular compartment shown here. When adapted to microfluidic systems to create a perfusable vasculature, this model might prove superior to study BBB function as well as disorders associated with dysfunctional cells within the BBB.

## 4 Experimental Section

### Fabrication of Micropillar Array 24‐Well Plate System and Agarose Microwell Culture Inserts Production

The 3D computer‐aided design (CAD) model of the micropillar array plate system in 24‐well plate format was prepared using the Solid Edge software (Siemens, Germany). Each well consists of 137 cylindrical micropillars (Ф 500 µm ×x H 700 µm; 350 µm spacing) as negative template to produce agarose culture inserts containing 137 microwells of the same dimension per insert. The plate system was fabricated from photopolymer RGD835 Vero white plus (Stratasys) via additive manufacturing technique using the Objet30 Prime polyjet printer available at the FabLab of KU Leuven (https://fablab‐leuven.be/). A total of 24 ring‐like removal tools were also fabricated to help removing inserts upon agarose hardening. To prepare the agarose microwell culture inserts, 550 µl of microwave‐heated 3% agarose solution (in sterile phosphate‐buffered saline (PBS)) was added to each well of the plate system that was pre‐sterilized with 70% isopropanol. The agarose solution was allowed to harden at room temperature (RT) for 5 min. The inserts were lifted gently from the plate system using the removal tools, and subsequently transferred to 24‐well plate under sterile condition. The inserts were immersed in 1 ml PBS and equilibrated overnight in the cell culture incubator prior to cell seeding.

### Differentiation of Human Embryonic Stem Cells (hESCs) into Cortical Neuroprogenitor Cells (NPC)

The H9 hESCs (WA09, purchased from the WiCell research Institute, female, RRID: CVCL_9773) colonies were maintained on matrigel‐coated 6‐well plates (Corning) in E8 flex medium [E8 basal medium (Gibco) supplemented with E8 supplement Flex and 5 U ml^−1^ Penicillin−Streptomycin]. The colonies were passaged twice a week (in 1:3 split ratio) using 0.5 mM EDTA (Gibco). To induce NPC, the hESCs were harvested into single‐cell suspensions by accutase (Sigma) and seeded at 2.5 million cells per well of 6‐well plate in the neural induction medium (NIM) consisting of neural maintenance medium (NMM) supplemented with the dual‐SMAD inhibitors SB431542 (10 µM, Tocris) and LDN193189 (1 µM, Miltenyi)^[^ ^34^ ^].^ (For NMM preparation, see Supporting Information: Extended Description of Methods.) At day 10, the neuroepithelial cells were passaged three times with Dispase II (Sigma) in the NMM up to 34 days to further purify the NPC. The generated DIV (*Day* In Vitro) 34 NPC were stored in liquid nitrogen cell bank for subsequent experiments. To enable cell viability tracking, a genome‐engineered H9 hESCs line bearing the constitutive tdTomato reporter expression cassette in the *Adeno‐Associated Virus Integration Site 1* (AAVS1) locus were used to generate NPC as described above. The use of hESCs was approved by the Human Ethics Committee at the University Hospital, Gasthuisberg, KU Leuven, Belgium.

### Medium‐Throughput Generation of Homogenous Size of NPC Spheroids and Neural Concentroids

The DIV34 NPC were thawed from liquid nitrogen and expanded in NMM as specified in each differentiation protocol. When confluent, the cells were harvested with accutase and seeded onto the agarose culture inserts at a density of 10000 NPC (DIV 46, after 12 days of 2D expansion of DIV34 NPC) per microwell to promote spheroid formation. For neural concentroids (NC) generation, approximately 137 spheroids were pooled together into a well of the 12‐well plate to generate one NC. In this case, the NMM was supplemented with basic fibroblast growth factor (bFGF), epidermal growth factor 2 (EGF2), brain‐derived neurotrophic factor (BDNF) and neurotrophin‐3 (NT3) as specified in the differentiation protocol to promote spheroid fusion and maturation under dynamic culture condition using an orbital shaker (75 round per minute). At defined time points, phase‐contrast images of the spheroids and NC were taken for the estimation of spheroid size using ImageJ software. For the tracking of spheroid viability, the tdTomato reporter expression was assessed via phase‐contrast fluorescence microscopy.

### Differentiation of Genome‐Engineered iETV2‐hiPSC into EC

A genome‐engineered iETV2‐hiPSC line available in the laboratory was used to generate EC.^[^ ^51^, ^84^ ^]^ Briefly, the iETV2‐hiPSC line was thawed from liquid nitrogen and the colonies were expanded in E8 flex medium on matrigel‐coated 6‐well plate. At 70% confluent, the cells was switched to NMM supplemented with doxycycline (5 µg ml^−1^) and bFGF to initiate endothelial differentiation by overexpression of the ETV2. After 2 days of differentiation, 2% fetal bovine serum (FBS) was added into the freshly prepared medium mixture and subsequently cultured for an addition 2 days. The cells were then harvested by accutase and subcultured (in 1:3 ratio) in NMM supplemented with doxycycline, bFGF, 2% FBS, and 2% human endothelial cell growth supplement (ECGS; Cell Applications Inc.) for another 4 days with medium changed every 2 days. The differentiated ECs were then used for co‐culture experiments or continued cultured for further analysis. The use of hiPSCs was approved by the Human Ethics Committee at the University Hospital, Gasthuisberg, KU Leuven, Belgium. The term pluripotent stem cells (PSCs) PSC is used to denote either hESCs or hiPSCs.

Generation of vascularized neural constructs using neural concentroids (NC) and eConcentroids (eNC). NC were generated as described above, whereas eConcentroids were generated by fusion of neural spheroids consisted of day 8 iETV2‐EC and DIV46 NPC at 1:1 ratio. On day 16 (Figure 6A), NC and eNC were embedded in 35 µl matrigel droplets with or without day 8 iETV2‐EC (1 million cells per matrigel droplet; *For procedures to embed NC and eNC in matrigel droplet, see Supporting Information: Extended Description of Methods*) and subsequently cultured statically in NMM supplemented with BDNF, NT3 and bFGF for 3 days. The constructs were then transferred onto a shaker for dynamic culture in BDNF and NT3‐supplemented NMM till day 30 to promote maturation. The constructs were then maintained in NMM up to day 70. At defined time points, brightfield images were taken and the migration distance was measured using ImageJ (*n* = 3, triplicate measurements) to obtain the migration speed for both NC and eNC embedded in matrigel without iETV2‐EC. On day 30 and 70, the samples were harvested for immunostaining and confocal microscopic analysis as described above.

### Generation of Vascularized Cerebral Organoid (vCO) and Co‐Culture with iETV2‐EC

For vCO generation, the hPSC and iETV2‐EC lines were mixed at 8:2 ratio, and non‐vascular COs were used as control. Briefly, both cell lines were dissociated into single cells using trypsin, and per each organoid, 20,000 cells, consisting of the given ratio of hPSC:iETV2‐hiPSC were resuspended in Neural Induction Medium (NIM) containing 50 µM Y27632. Hanging‐drop method was used for aggregate formation. Drops with 30 µl of NIM embracing the desired cell density were laid into the lid of the culture dish and incubated for 24 hours. The day after, named day 1, formed aggregates were transferred to the non‐treated suspension culture dishes and incubated on an orbital shaker until the end of the experiment. The aggregates were cultured in NIM containing dual SMAD inhibition small molecules until day 7. Afterward, they were treated with Neural Proliferation Medium (NPM) consisting of bFGF and EGF until day 25, and from day 25 onwards they were cultured in Neural Maturation Medium (NMM) with BDNF and GDNF as neurotrophic factors. The media were changed every other day. To induce basal activation of ETV2 expression, 2 µg ml^−1^ doxycycline was added to media on day 4, and 5 µg ml^−1^ doxycycline were continuously added from day 7 to keep the ETV2 activation. On day 10, the CO and vCO were embedded in the matrigel droplets, or co‐cultured with iETV2‐EC containing matrigel droplets. Each organoid was embedded in 30 µl matrigel containing 100,000 iETV2‐EC and transferred to a 37 °C incubator for 30 min to induce matrigel polymerization. The embedded organoids continued to grow and were induced with 5 µg ml^−1^ doxycycline treatment in NPM followed by NMM.

### Immunofluorescence Staining and Confocal Microscopy

Cells in monolayer cultures were fixed with 4% paraformaldehyde (PFA) for 20 min at RT, whereas spheroids and concentroids harvested at defined time points were fixed with 4% paraformaldehyde (PFA) overnight at 4 °C. Subsequently, the samples were immersed in 30% sucrose solution for 24 h before embedded in OCT compound by snap freezing in liquid nitrogen bath. Cryosections of 10 µm thickness were made using a cryostat (Cryostar NX70, Thermo Scientific), and the slides were stored at −20 °C until analysis. After washing with PBS, the cells or cryosections were blocked and permeabilized with 0.1% Triton X‐100 in PBS (PBST) containing 5% normal goat serum (Dako) for 20 min at RT. Diluted primary antibodies were added to the samples and incubated overnight at 4 °C in humidified chambers (Table S11). Secondary antibodies (species‐matched Alexa Fluor 405, 488, 555, and 647 antibodies; ThermoFisher scientific) were diluted in the Dako REAL Antibody diluent and added to the samples for 45 min at RT. After washing with PBS, the nuclei were stained with Hoechst 33 342 (Sigma, 1:1000 dilution), washed, and mounted with ProLong Gold antifade mountant (Life technologies). For 3D confocal imaging of whole spheroid, the durations for immunostaining were doubled to ensure sufficient penetration of the primary and secondary antibodies as well as washing to increase signal‐to‐noise ratio. The samples were treated with RapidClear clearing agent (Sunjin Lab, Taiwan) and confocal imaging were performed using the Nikon C2 confocal microscope equipped with long‐distance objective lens.

### Endothelial and Neuronal Network Formation Assay

1×10^6^ DIV46 NPC was resuspended with day 8 iETV2‐EC (1:1 ratio) in 35 µl matrigel and incubated in cell culture incubator for 1 hour to allow polymerization. The co‐culture droplets were cultured statically in NMM supplemented with doxycycline (5 µg ml^−1^) using ultra‐low attachment 12‐well plate (Corning). The medium was refreshed every two days, and the samples were harvested on days 7 and 21 days for fixation in 4% PFA overnight at 4 °C, washed with PBS, and permeabilized with 0.1% Triton X‐100 in PBS for 1 h. The samples were stained with primary antibodies against human beta III‐tubulin (TUJ1), platelet endothelial cell adhesion molecule/cluster of differentiation (CD31), and glial fibrillary acidic protein (GFAP), labeled with species‐matched Alexa Fluor secondary antibodies, and optically‐cleared with RapidClear clearing agent for whole construct 3D confocal imaging using the Nikon C2 confocal microscope as described above.

### Quantification of CD31 and VE‐CAD Expression by iETV2‐ECs via Flow Cytometry

After 8, 21, and 35 days of endothelial differentiation from iETV2‐hiPSC, the iETV2‐ECs (n = 3) were harvested with accutase and resuspended into single‐cell suspension in medium supplemented with 10% FBS to inactive the accutase by incubation at room temperature for 6 min. After washing with ice‐cooled FACS buffer (PBS with0.1% BSA), the cell pellets were stained with anti‐human CD31 PE‐Cyanine7 antibody (1:200 dilution; Biolegend) and anti‐human VE‐Cadherin biotin antibody (VE‐CAD; 1:200 dilution; ThermoFisher Scientific) or VE‐CAD APC antibody (1:200 dilution; Biolegend) for 30 min at 4 °C. In case the biotin‐conjugated VE‐Cadherin was used, the secondary staining step was performed with Streptavidin‐APC (1:500 dilution; ThermoFisher Scientific) antibody or a Streptavidin‐FITC (1:500 dilution; ThermoFisher Scientific). After each staining steps, cells were washed with FACS buffer. Unstained and single‐stained cells were included as controls. Flow cytometry was performed at the FACS core facility of KU Leuven using the BD FACSymphony flow cytometer. Between 10,000 and 100,000 events were recorded for each sample, and the data was analyzed using FlowJo software (FlowJo).

### Whole‐Cell Patch Clamping on Agarose‐Embedded Concentroid Section

Neural concentroids were harvested on day 180 and immediately embedded in 2% low‐melting agarose (Sigma–Aldrich (A0576)) diluted in PBS: 137 mM NaCl, 2.7 mM KCl, 10 mM Na_2_PO_4_, 1.8 mM KH_2_PO_4_, pH = 7.4). Agarose was dissolved using microwave and kept in a water bath at 40 °C until use. Embedding was done on ice to quickly solidify the agarose gel and subsequently sectioned using a Leica VT1200S vibratome in ice‐cold oxygenated (95% O_2_ and 5% CO_2_) artificial cerebrospinal fluid (acsf) solution, containing: 127 mM NaCl, 25 mM NaHCO_3_, 1.25 mM NaH_2_PO_4_, 2.5 mM KCl, 1 mM MgCl_2_, 2 mM CaCl_2_, and 25 mM glucose. Afterward, sections were stored in acsf at room temperature. For recordings the slices were continuously perfused in a submerged chamber (Warner Instruments) at a rate of 3–4 ml min^−1^ with oxygenated acsf at a temperature 33 ± 1 °C. Internal whole cell patch solution contained: 135 mM K‐gluconate, 4 mM KCl, 10 mM HEPES, 4 mM Mg‐ATP, 0.3 mM Na2‐GTP, 10 mM phosphocreatine (pH = 7.3, ≈295 mOsm). The slices were recorded using a Multiclamp 700B amplifier and data was analyzed using Clampfit 10.7 (Axon Instruments). Currents were recorded at 20 Hz and low‐pass filtered at 3 kHz when stored. Pipettes were pulled using a Sutter P‐1000 and resistance ranged from 3 to 5 MΩ. The series resistance was compensated to 70–75%. The I/V‐traces were generated using a step protocol (−70 to +20 mV, 10 mV steps) from a holding potential of −70 mV. Active and inactive currents were measured at the beginning and end of the voltage steps, respectively. Resting membrane potential (Vm) was measured after the establishment of whole‐cell configuration (in current clamp, Iholding = 0 pA).

### Gene Expression Analysis by Reverse‐Transcriptase Quantitative Polymerase Chain Reaction (RT‐qPCR)

The samples were harvested in TRIzol KS reagent (Life Technologies) and the total RNA was extracted by phase‐separation with chloroform followed by RNA isolation and purification using the RNeasy kit (Qiagen) according to the manufacturer instructions. The total RNA content of each sample was quantified using NanoDrop system (Thermofisher Scientific, USA), and the cDNA was synthesized using the SuperScript III First‐Strand Synthesis System kit (Thermofisher). The RT‐qPCR analysis (*n* = 3; duplicate measurements) was performed on the ViiA 7 Real‐Time PCR System (Applied Biosystems, USA) using Platinum SYBR Green qPCR Supermix‐UDG with ROX (Invitrogen, CA, USA) and the primer sequences were listed in Table S12. Gene expression (cycle threshold, CT) values were normalized to the housekeeping gene GAPDH (Glyceraldehyde‐3‐phosphate dehydrogenase) and the delta CT (dCT) values were calculated.

### Bulk RNA Sequencing Analysis

Total RNA extracted from iETV2‐EC samples harvested on days 8, 14, and 21 days (*n* = 3) were prepared and sequenced single‐end on NovaSeq SP 100×6 bp resulting in an average of 10 million reads per sample. All reads were trimmed by removing poor‐quality base pair (bp), poly N tails, ribosomal RNA, and adaptors. Quality controls were performed before and after trimming using AfterQC and FastQC followed by mapping every read to the reference human genome GRCH38 (release 81.2) using STAR.^[^ ^85^ ^]^ Assembly of all mapped reads was performed using python package StringTie^[^ ^86^ ^]^ generating raw gene counts for further downstream analysis.

### Integration of Publicly Available Bulk RNA Sequencing Data for DESeq2 and Principal Component Analysis (PCA)

In a total of 4100 samples (comprised of 141 studies) of human endothelial RNA sequencing database available from the Massive Mining of Publicly Available RNAseq Data from Human and Mouse (ARCHS4) platform was downloaded using the data extraction script provided by the ARCHS platform in RStudio.^[^ ^87^ ^]^ The sample metadata were then retrieved to identify datasets that contain relevant cell types and characteristics. This resulted in a refined dataset of 320 samples (Table S13) which was then integrated with the iETV2‐EC RNA sequencing dataset by aligning the genes and after removing any duplicate gene datasets. Differential gene expression analysis was performed using DESeq2 package whereby genes with less than 5 reads were filtered out. Standardization of the integrated data frame was then performed by the variance stabilizing transformation (VST) method.^[^ ^88^ ^]^ PCA and normalized gene count plots were performed via ggplot functions. Heatmaps and Pearson correlation plots were created via pheatmap and corrplot functions using the transformed data.

### Sample Homogenization, Single Nuclei Isolation, Library Preparation and Sequencing

For nuclei isolation, all following steps were performed on ice with pre‐cooled buffers, and centrifugation steps were performed at 4 °C. In brief, the snap‐frozen samples were thawed using 500 µl ice‐cold homogenization buffer [HB; consisting of 320 mM sucrose, 5 mM CaCl_2_, 3 mM magnesium acetate, 10 mM Tris pH 7.8, 0.1 mM EDTA, 0.1% NP40, 1X complete protease inhibitor (Roche), 1 mM β‐mercaptoethanol and RNase‐In inhibitor (Promega)] for 5 min to prevent the nuclei from rupturing. The samples were then homogenized with pestle A (5 gentle strokes) and pestle B (15 gentle strokes) and incubated on ice for 5 min. The homogenates were filtered using Flowmi 70 µm cell strainers and the filtrates were centrifuged at 500 g for 5 min. The resulting pellets were resuspended in 2.65 ml washing buffer (HB without NP40) and 2.65 ml gradient medium (5 mM CaCl_2_, 50% Optiprep, 3 mM magnesium acetate, 10 mM Tris pH 7.8, 1X complete protease inhibitor, 1 mM β‐mercaptoethanol and RNase‐In inhibitor) and the resulting solution was added gently on top of a cushion solution (29% Optiprep, 150 mM KCl, 30 mM MgCl_2_, 60 mM Tris pH 7.8 and 250 mM sucrose) for centrifugation at 7700 rpm for 30 min at 4 °C. The nuclei pellets were resuspended in 25 µl resuspension buffer (1X PBS, 2% BSA), filtered using Flowmi 40 µm cell strainers, and performed nuclei counting using LUNA automated cell counter (Logos Biosystems) according to the manufacturer's instructions. Subsequently, the diluted nuclei suspensions were loaded onto the 10X Chromium Single Cell Platform (10X Genomics) (Next GEM Single Cell 3′ library and Gel Bead Kit v3.1) according to the manufacturer's protocol (10x User Guide; CG000204, Revision D). Generation of gel beads in emulsion (GEMs), barcoding, GEM‐RT cleanup, complementary DNA amplification, and library construction were all performed according to the manufacturer's protocol. Individual sample quality was assessed using a Tapestation (Agilent). Qubit 2.0 (ThermoFisher Scientific) was used for library quantification before pooling libraries. The final library pool was sequenced on NextSeq2000 (Illumina) instrument using NextSeq 1000/2000 P3 kit v3 for 2 lanes of 100‐base‐pair paired‐end reads.

### Single Nuclei RNA Sequencing (snRNAseq) Analysis

The Cell Ranger version 6.1.1 was used to process, align, and summarize unique molecular identifier (UMI) counts against the 10X Genomics pre‐built human GRCh38 reference genome datasets (2020‐A, July 7, 2020). Each sample was processed using the semi‐supervised machine learning classifier – Debris Identification using Expectation Maximization (DIEM)^[^ ^89^ ^]^ to eliminate debris and empty droplets. More specifically, Debris and test sets were specified for droplets using set debris_test_set(sce, min_counts = 200, min_genes = 100), and genes with no expression were filtered out with filter_genes(sce, cpm_tresh = 10). Cell type clustering was initialized by performing PCA analysis using get_pcs(sce, n_var_genes = 2000, min_genes = 200, n_pcs = 30, threads = 8, seedn = 1234) and k‐means algorithm using init(sce, k_init = 20, nstart_init = 30, min_size_init = 10, threads = 8). We run expectation maximization algorithm run_em(sce, threads = 8, model = “DM”) to estimate the parameters of the multinomial mixture model and assign clusters for each droplet in the test set using assign_clusers(sce). We removed clusters that were assigned as debris using the command function call_targets(sce, clusters = “debris”, thresh_score = NULL, min_genes = 0). Further downstream analyses were performed using Seurat analysis pipeline provided by the VIB Bioinformatics Core, Belgium. Annotation of the cell clusters was done by directly referencing to the online interactive single cell portal of human brain organoid databases^[^ ^57^ ^]^ (https://singlecell.broadinstitute.org) as well as by integrating our datasets into the reproduced and annotated seurat objects of the publicly available databases for human brain organoids, fetal and adult human brain cortex as mentioned above using Seurat‐integration package. The online g:Profiler platform (https://biit.cs.ut.ee/gprofiler/gost) was used to perform functional enrichment analysis of the identified top‐20 highly variable gens (HVGs) for ETV2plus NCs and NCs.

### RNA Velocity Analysis with scVelo, and Lineage Trajectory Inference by Slingshot

The Python package of scVelo^[^ ^58^ ^]^ and its accompanying software Velocyto^[^ ^90^ ^]^ were used to perform RNA velocity analysis on the snRNAseq data of the merged seurat object for ETV2plus NCs and NCs, according to the online scripts provided by Bergen et al. with modifications (https://scvelo.readthedocs.io/). The matrix for spliced and unspliced transcripts was constructed using the velocyto command line tool that works directly with the cellranger output directory, and the human reference.gtf annotation and mask repeat files were used. For the trajectory inference, *Slingshot* ^[^ ^64^, ^65^ ^]^ trajectories analysis was performed on the merged Seurat object of ETV2plus NCs and NCs. Next, cells were binned (15 bins) and mixed ANOVA tests with Benjamini‐Hochberg correction were used to identify differential regulon activity along the pseudotime axis (based on AUC score), comparing the identified six lineage trajectories.

### VoxHunt Analysis

The online VoxHunt^[^ ^61^ ^]^ analysis script (https://quadbiolab.github.io/VoxHunt/index.html) was performed to obtain in silico visualization of spatial similarity patterns to resolve regional identity of our snRNAseq datasets for ETV2plus NCs and NCs by mapping to the developing mouse (in situ hybridization (ISH) data from the Allen Developing mouse brain atlas; https://developingmouse.brain‐map.org/) and human brain (bulk RNAseq data from BrainSpan; https://www.brainspan.org/) databases. Briefly, the Allen Brain Atlas (ABA) gene expression data for mouse brain with annotated brain structures at E11.5 – E18.5 and P4 – P56 were used to extract top‐10 regional marker genes for each developmental stages to facilitate the projection of our snRNAseq datasets for estimation of regional similarity and for 3D mapping. To compare to human brain data, the RNAseq data of microdissected human brain regions from BrainSpan and the top‐10 regional marker genes extracted from the ISH mouse data were used. To estimate regional proportions in ETV2plus NCs and NCs, our snRNAseq data was deconvoluted based on the selected regional markers obtained from the ABA ISH reference mouse brain data at E18.5, P4, and P14 respectively.

### pySCENIC Analysis

The Single‐Cell Regulatory Network Inference and Clustering (pySCENIC^[^ ^63^ ^]^) analysis was performed in a conda environment installed with Python and pySCENIC package and its associated dependencies, according to the scripts as described by Van de Sande et al. The auxiliary files containing the list of TFs, ranking databases, and motif annotations for human were downloaded from the respective depository (https://resources.aertslab.org/cistarget/). The expression matrix and metadata of the merged seurat object of ETV2plus NCs and NCs were extracted to create the loom file, and the GRNBoost2 network inference algorithm was performed to infer gene regulatory network and to generate the co‐expression modules. Regulon prediction aka cisTarget algorithm was performed to generate a list of adjaciences between a TF and its targets, thus identified regulons specific to each cluster based on the calculated regulon specificity score (RSS). Following the calculation of cellular enrichment (aka AUCell) values, the online Scope visualization tool (http://scope.aertslab.org) was used to explore the results. Functional enrichment analysis of the identified regulons was performed via the online g:Profiler platform. The regulon gene lists were then used to map out the gene regulatory networks for the enriched TFs and their target genes via iRegulon tool (http://iregulon.aertslab.org/index.html) in Cytoscape software (Recovery: enrichment score threshold = 3, ROC threshold for AUC calculation = 0.03, rank threshold = 5000; TF prediction: minimum identity between orthologous genes = 0, maximum false discovery rate (FDR) on motif similarity = 0.001).

### Statistical Analysis

All experiments were performed in triplicate or as stated in the figure legends, and the results are presented as the mean ± s.d, except for electrophysiology measurement the results are presented as the mean ± SEM. Statistical significance was evaluated using two‐tailed Student's *t*‐tests for two‐group comparisons and one‐way ANOVA with Tukey's post hoc test for multiple‐group comparison, and two‐way ANOVA with Sidak's post hoc test for cell migration speed assay. *P* < 0.05 was considered to be significant.

## Conflict of Interest

The authors declare no conflict of interest.

## Author Contributions

Y.C.C., S.K.T, S.S., S.Z., K.W., B.K.V., and S.V. designed and performed the experiments, analyzed and/or interpreted the data; R.R., Y.Z., K.A., and A.L. designed and/or performed the experiments; Y.C.C., C.V., V.P., and L.A.vG conceived and/or supervised the project, designed and analyzed and interpreted the data. The manuscript was mainly written by Y.C.C. and C.V. with contributions from all of the authors.

## Supporting information

Supporting Information

Supporting Table 1

Supporting Table 2

Supporting Table 3

Supporting Table 4

Supporting Table 5

Supporting Table 6

Supporting Table 7

Supporting Table 8

Supporting Table 9

Supporting Table 10

Supporting Table 11

Supporting Table 12

Supporting Table 13

## Data Availability

The main data supporting the findings of this study are available within the paper and its Supplementary Information. The datasets from the quantitative analyses generated during this study, including source data and the data used to make the figures as well as all images, are too large and numerous to be publicly shared, but they are available for research purposes from the corresponding authors on reasonable request. Both bulk RNAseq and snRNA seq data have been deposited in the NCBI Gene Expression Omnibus (GEO), with accession number GSE192919 and GSE233741, respectively.
