# Supplementary material for: Spatially Self‐Organized Three‐Dimensional Neural Concentroid as a Novel Reductionist Humanized Model to Study Neurovascular Development
Source: Adv Sci (Weinh). 2023 Nov 30;11(5):2304421. doi: 10.1002/advs.202304421 (PMC10837345; doi:10.1002/advs.202304421)
Supplement: Supplementary file 1 — Supporting Information [file ADVS-11-2304421-s006.pdf]

ADVANCED SCIENCE
Open Access

Supporting Information

for *Adv. Sci.*, DOI 10.1002/advs.202304421

Spatially Self-Organized Three-Dimensional Neural Concentroid as a Novel Reductionist Humanized Model to Study Neurovascular Development

Yoke Chin Chai, San Kit To, Susan Simorgh, Samantha Zaunz, YingLi Zhu, Karan Ahuja, Alix Lemaitre, Roya Ramezankhani, Bernard K. van der Veer, Keimpe Wierda, Stefaan Verhulst, Leo A. van Grunsven, Vincent Pasque and Catherine Verfaillie**

Supporting Information

Title:

Spatially Self-Organized Three-Dimensional Neural Concentroid As A Novel Reductionist Humanized Model To Study Neurovascular Development

Yoke Chin Chai[1], San Kit To[2], Susan Simorgh[1], Samantha Zaunz[1], YingLi Zhu[1], Karan Ahuja[1], Alix Lemaitre[1], Roya Ramezankhani[1], Bernard K. van der Veer[3], Keimpe Wierda[4], Stefaan Verhulst[5], Leo A. van Grunsven[5], Vincent Pasque[2], Catherine Verfaillie[1*]*

<u>List of Tables:</u>

<u>Table 1:</u> The Top-20 highly variable genes (HVGs) identified for ETV2plus NCs and NCs based on snRNAseq analysis.

<u>Table 2:</u> The identified gene markers for each cluster obtained based on the UMAP plot of the merged seurat object of ETV2plus NCs and NCs.

<u>Table 3:</u> The RNA velocity pseudotime obtained via PAGA analysis.

<u>Table 4:</u> Cluster-specific putative driver genes identified via RNA velocity analysis.

<u>Table 5:</u> The ranked velocity genes per cluster obtained via PAGA analysis.

<u>Table 6:</u> Table of adjaciencies showing the identified transcription factors, their target genes and importance for ETV2plus NCs vs. NCs, and between C3, C4, C7 and C8 clusters.

<u>Table 7:</u> Table showing the regulon specificity score (RSS) for regulons identified for ETV2plus NCs vs. NCs.

<u>Table 8:</u> Genes change between start_end per lineages (progenitor markers for C2 cluster).

<u>Table 9:</u> Table showing the regulon specificity score (RSS) for regulons identified for cluster C3, C4, C7 and C8.

<u>Table 10:</u> Metadata of pySCENIC analysis for *ATF3_(+)*, *ETS1_(+)* and *PRRX2_(+)* regulons, and calculation of percentage of cells based on the Area Under the Curve (AUC).

<u>Table 11:</u> List of primary antibodies used for immunostaining.

<u>Table 12:</u> List of primer sequences used for RT-qPCR. fw = forward sequence; rv = reverse sequence.

<u>Table 13:</u> List of accession numbers, cell types, and source/culture conditions for the publicly available RNA sequencing data used in the DESeq2 analysis.

List of Supplementary Figures

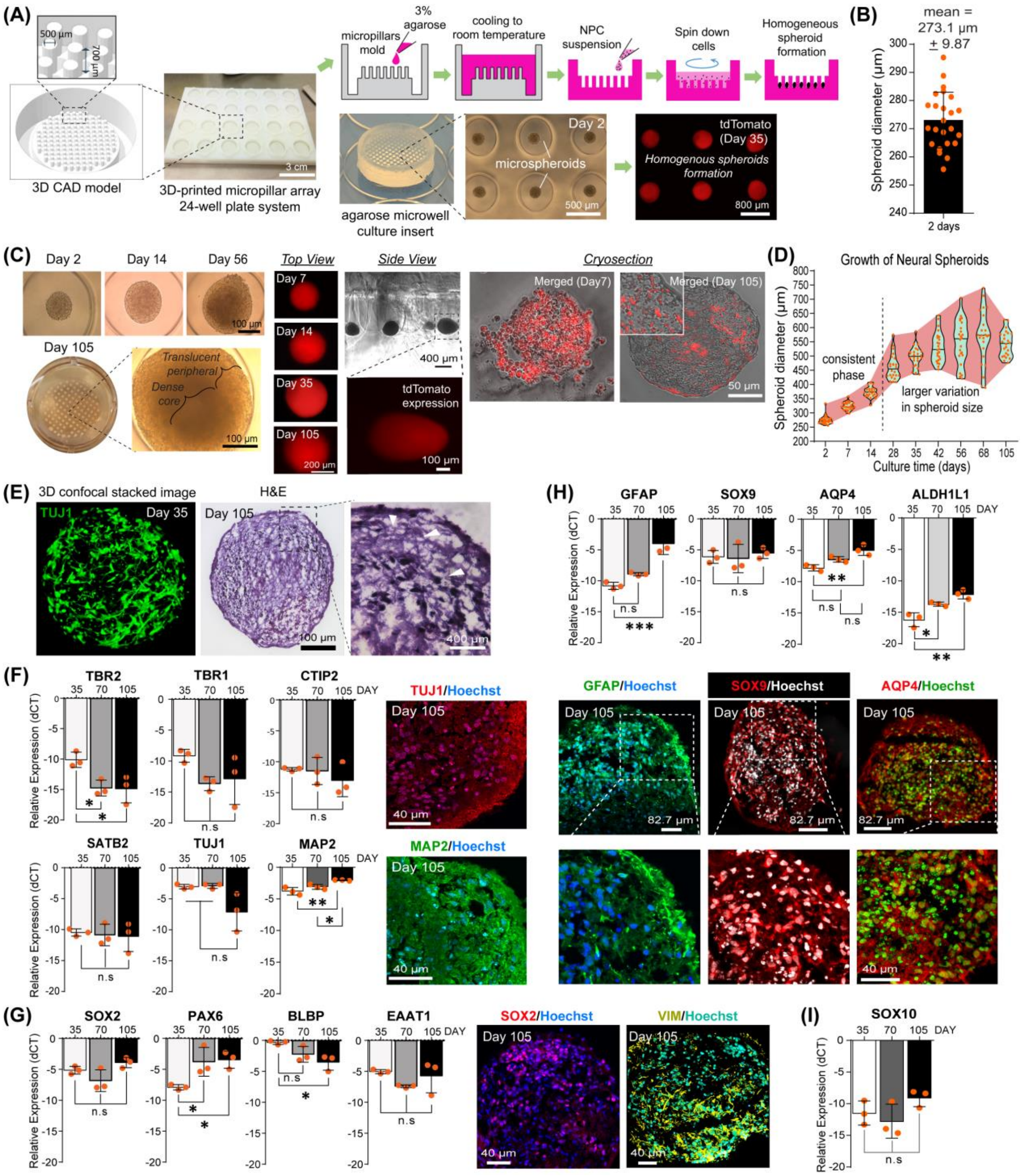

<u>Figure S1:</u> Medium-throughput generation of neural spheroids and their progression into a mixed population of neuronal and astroglial cells. **(A)** The CAD model and 3D-printed micropillar array 24-well plate system as negative mold to produce 24 agarose culture inserts (each containing 137 microwells; diameter x height = 500 µm x 700 µm), and the schematic of agarose culture insert production and spheroid generation (10,000 NPCs per spheroid). In total 3288 NPC spheroids could be generated per batch of 24 agarose culture inserts. **(B)** Measured spheroid diameter = 273.1 µm <u>+</u> 9.87 on 2 days after seeding, with <10 µm variation (n = 24

spheroids). **(C)** Representative images of self-aggregated neural spheroids in the microwells and cell viability tracking by live cell imaging and cryosectioning for the expression of genetically-encoded tdTomato reporter by the neural spheroids over 105 days of culture. **(D)** Growth of spheroids over time in culture (n = 24 spheroids). **(E)** 3D confocal stacked image of optically-cleared day 35 and hematoxylin and eosin (H&E) staining of neural spheroids at day 105 showing TUJ1$^+$ neurite network formation (white arrow heads). **(F, G, H, I)** RT-qPCR analysis (Day 35, 70, 105; n = 3 agarose culture inserts, each insert containing 137 spheroids (i.e., 3 x 137 = 411 spheroids per time point; each insert was analyzed in triplicate) and immunofluorescence staining (Day 105) for representative markers of cortical neurons [upper and deep layer neurons: *TBR2*, *TBR1*, *CTIP2*, *SATB2*; general neuronal markers: *TUJ1*, *MAP2*); ***F***], radial glia (*SOX2*, *PAX6*, *BLBP*, *EAAT1*, *VIM*; ***G***), astrocytic cells (*GFAP*, *SOX9*, *AQP4* and *ALDH1L1*; ***H***) and oligodendrocytes (*SOX10*; ***I***), respectively. All data shown are mean $\pm$ s.d.; one-way ANOVA with Tukey's multiple comparisons test: *p<0.05, **p<0.01, ***p<0.001, n.s = not significant. Nuclei were counterstained with Hoechst.

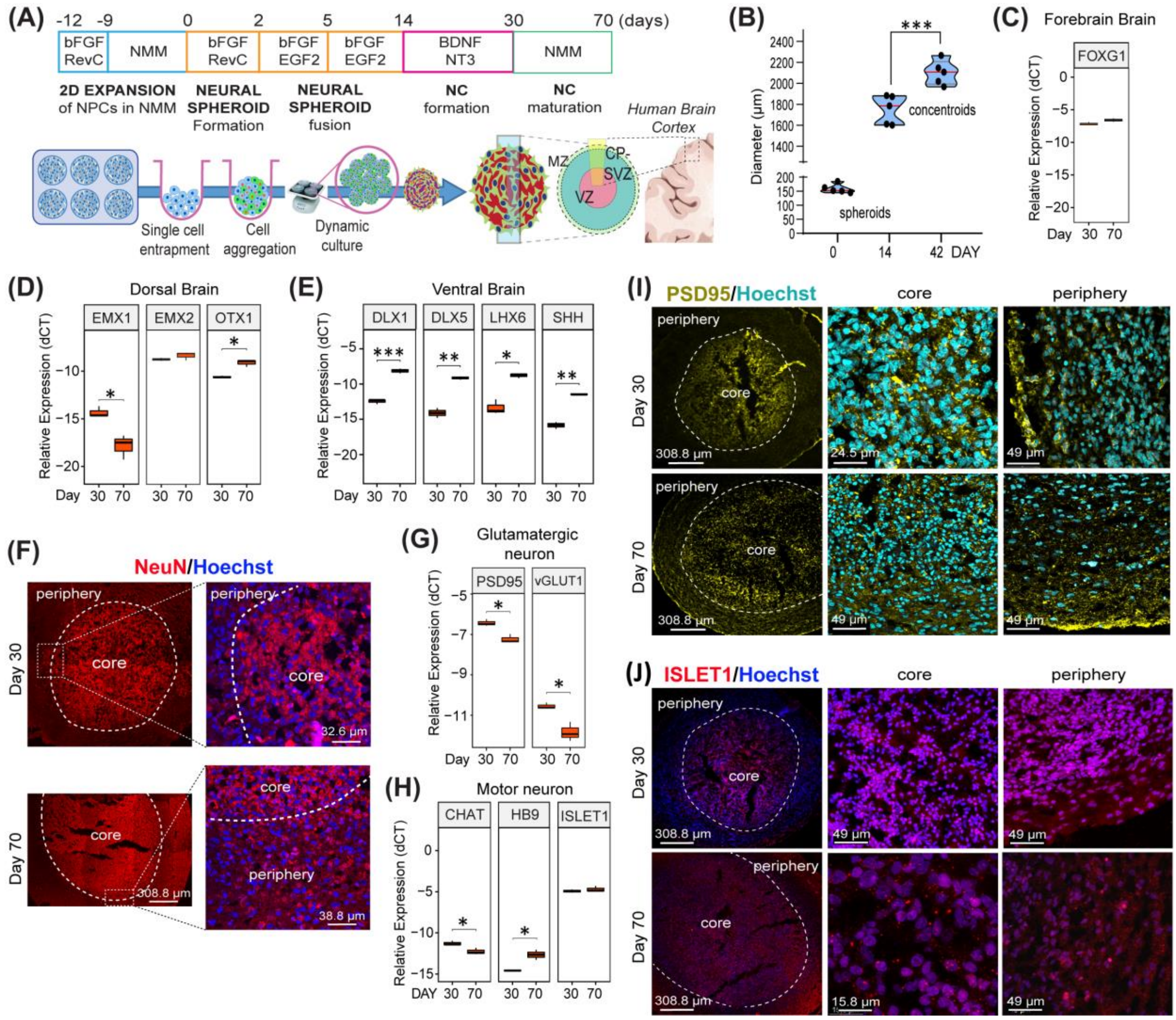

<u>Figure S2:</u> Characterization of forebrain identity and neuronal subtypes within the neural concentroids. (A) Schematic of culture protocol to promote neural spheroid fusion into concentroids and subsequent maturation by placing ~130 spheroids in each well of a 12-well plate on an orbital shaker (at 75 round per minute). **(B)** Diameter of concentroids up to 42 days as compared to starting spheroids (n = 5). **(C, D, E, G, H)** RT-qPCR analysis of representative gene markers for forebrain, dorsal and ventral identity, glutamatergic, and motor neuronal subtypes on day 30 and 70. All data shown are mean $\pm$ s.d. (n = 3, duplicate measurements; unpaired t-test compared to day 30 (*p<0.05, **p<0.01, ***p<0.001). **(F, I, J)** Immunofluorescence images showing staining for neuronal marker NeuN, glutamatergic (PSD95), and motor (ISLET1) neurons within the neural concentroids on day 30 and 70. Nuclei were counterstained with Hoechst.

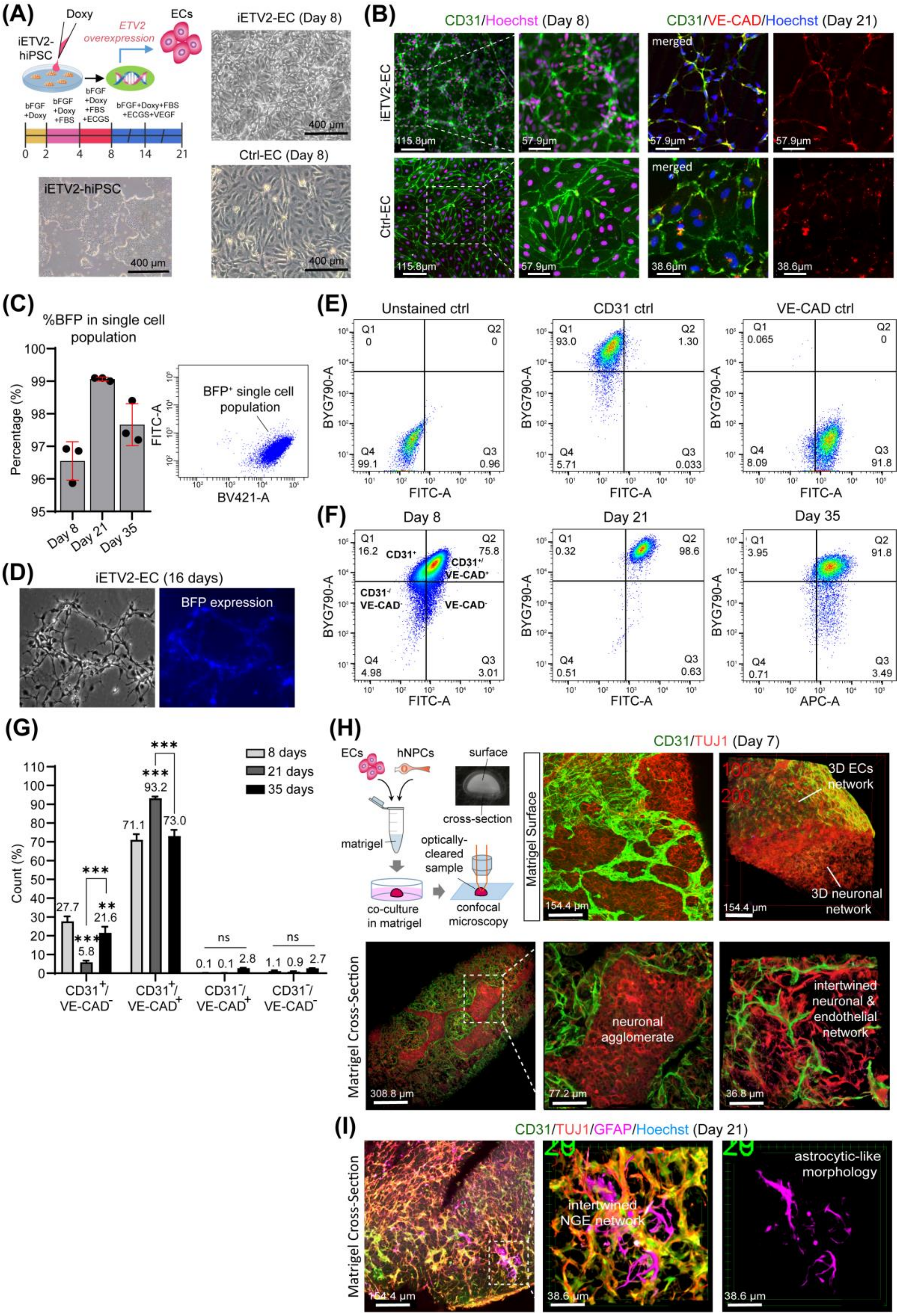

Figure S3: ETV2 overexpression in neurogenic medium produced EC and formed intertwined neuronal & endothelial network when co-cultured with NPCs. (A) Schematic of EC differentiation from the iETV2-hiPSC line by doxycycline induction. Representative

brightfield images of the iETV2-hiPSC colonies, and the iETV2-EC generated in NMM and the control EC (Ctrl-EC; based on liver differentiation medium) on day 8**. (B)** Immunostaining of iETV2-EC and Ctrl-EC on day 8 and day 21 for CD31 and VE-CAD. Nuclei were counterstained with Hoechst. **(C, D)** Flow cytometry quantification of the percentage of BFP^+ cells (from genetically encoded BFP reporter) on day 8, 21 and 35 days (>95% for all time points; n = 3), and representative images of iETV2-EC culture morphology and BFP expression on day 16. **(E, F)** Representative flow cytometry dot plots showing the gating strategy (starting from single BFP^+ gated cells) used to quantify the CD31 and VE-CAD expression for the day 8, 21 and 35 iEVT2-EC cells and theunstained and single-stained control cells. (**G**) Flow cytometry quantification of CD31 and VE-CAD surface markers on iETV2-EC day 8, 21 and 35 (n = 3). **(H)** Co-culture of iETV2-EC with NPC in matrigel for 7 days showing $CD31^+$ endothelial networks surrounding and intertwined with $TUJ1^+$ neuronal cells 7 and 21 days after co-culture. **(I)** Immunostaining for CD31, TUJ1 and GFAP of iETV2-EC and NPC co-cultures on day 21 showing intertwined neuro-astroglial-endothelial (NGE) network.

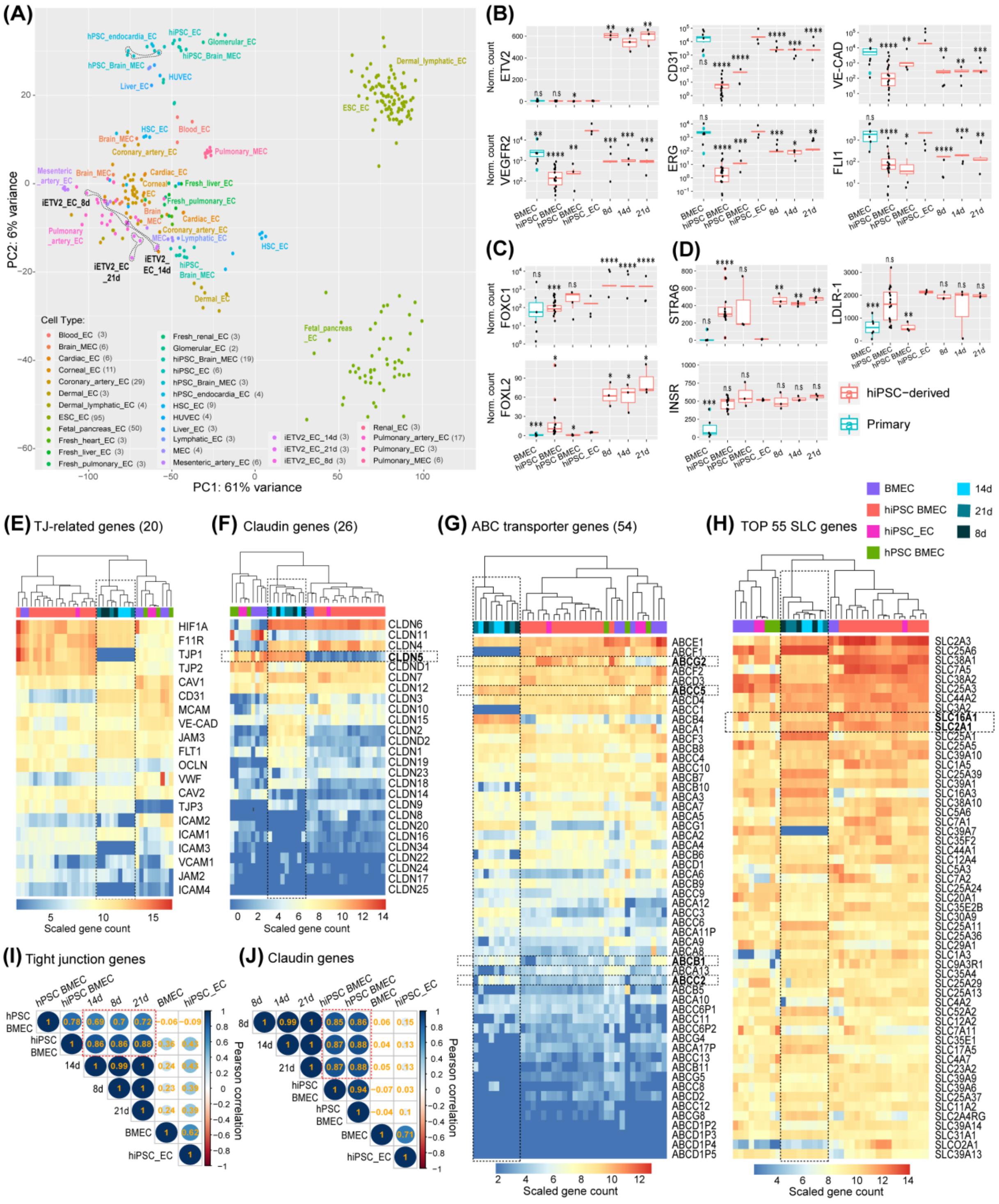

<u>Figure S4:</u> ETV2 overexpression in neurogenic medium produced EC with brain endothelial-like phenotypes. (A) Principal component analysis (PCA) plot of body-wide ECs, hiPSC-EC, ESC-EC, hiPSC- and hPSC-derived MEC or brain MEC (BMEC), as well as iETV2-EC. The iETV2-EC on day 8, 14 and 21 days (delineated by dotted lines) clustered among some body-wide EC isolated from human donors, the MEC and BMEC, but separated in distant from the hiPSC-EC, HSC-EC, ESC-EC, dermal lymphatic EC and fetal pancreas EC. Number in brackets next to the legend of each EC type indicates the number of sample data

used for the analysis. The accession numbers, cell types and source/culture condition for each sample are listed in Table 3. **(B, C, D)** Box plots showing normalized counts between the different EC types for the endothelial gene markers *ETV2*, *CD31*, *VE-CAD*, *VEGFR2*, *ERG* and *FLI1*, the putative BBB endothelial transcription factors *FOXC1* and *FOXL2*, and the retinol-binding protein (*STRA6*), insulin receptor (*INSR*) and low-density lipoprotein receptor-1 (*LDLR-1*). Unpaired t-test as compared to hiPSC_EC: *$p<0.05$, **$p<0.01$, ***$p<0.001$, ****$p<0.0001$, n.s = not significant. **(E, F, G, H)** Heatmaps of scaled gene counts for tight junction (TJ; D), claudin family (E), the ABC transporters (F) and the top-55 highly expressed solute carriers family (SLC; G) by the primary BMECs, hiPSC- and hPSC-derived BMECs, hiPSC-EC and iETV2-EC generated on day 8, 14 and 21. **(I, J)** Pearson correlation analysis showing high correlation of the transcriptomic profiles for TJ and CLDN gene markers between iETV2-ECs and hiPSC- and hPSC-derived BMEC, and low correlation to that of hiPSC-EC.

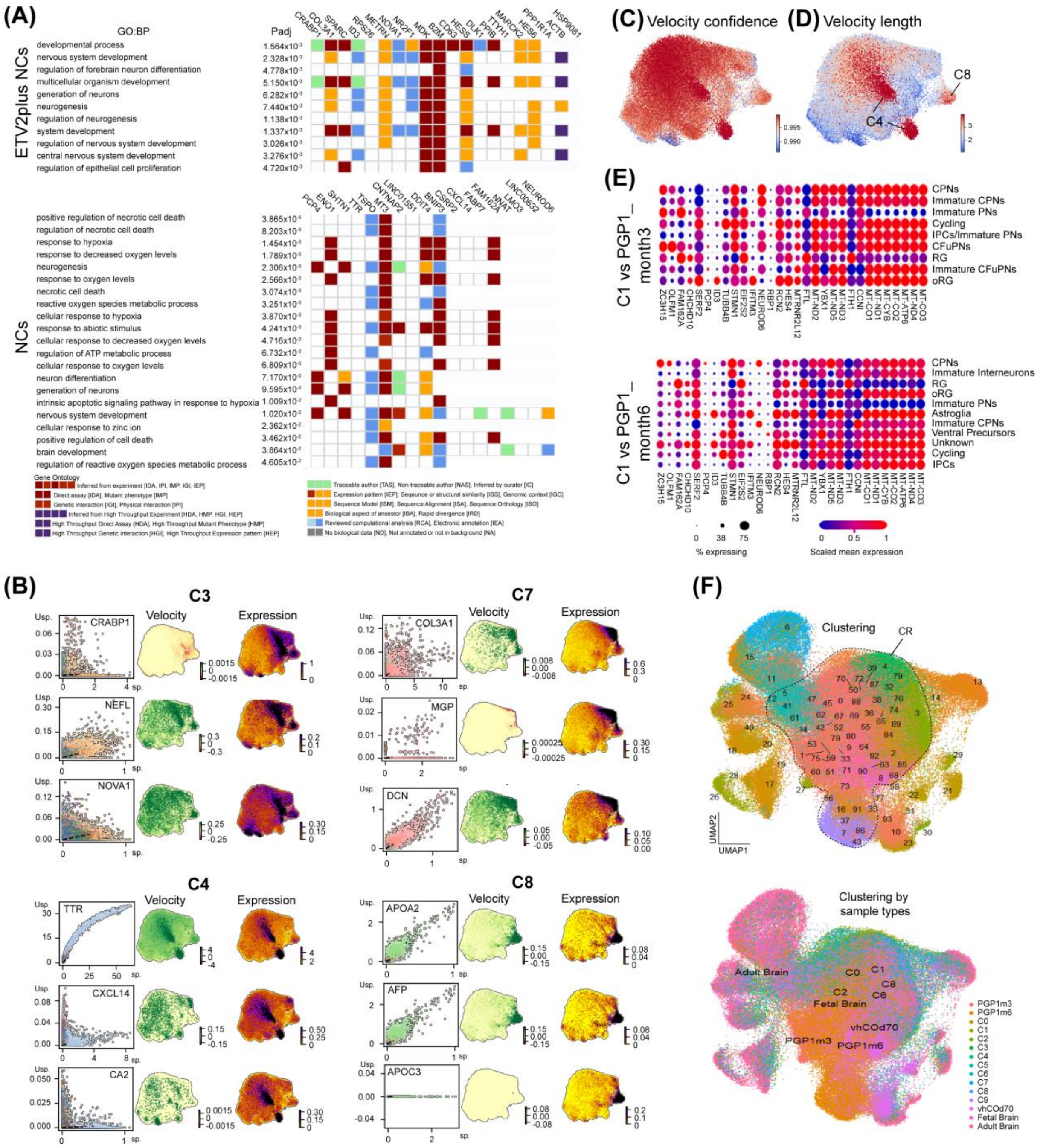

Figure S5: **(A)** Functional enrichment analysis of the top-20 highly variable genes (HVGs) in ETV2plus NCs and NCs via g:Profiler showing ETV2plus NC was enriched with pathways mainly associated to nervous system development, whereas NC was enriched with pathways mainly associated to necrotic cell death and hypoxia responses. **(B)** RNA velocity analysis of top-3 gene markers specific to cluster 3, 4, 7 and 8. (Left panel) Dot plots showing the unspliced (Usp.) and spliced (sp.) ratio of each gene marker per cell per cluster. (Middle and right panels) Heatmaps showing RNA velocity and the expression of each gene marker per cell per cluster. **(C, D)** UMAP plot showing high velocity confidence was estimated throughout the merged Seurat object of ETV2plus NCs and NCs; UMAP plot showing cluster 4 (C4) had the highest

velocity length. **(E)** Dotplots obtained by referencing the top-30 marker genes of C1 cluster to the online interactive single cell portal of human brain organoid databases, i.e., PGP1_month 3 and month 6 (https://singlecell.broadinstitute.org). oRG = outer radial glia; RG = radial glia; IPCs = intermediate progenitor cells; CFuPNs = corticofugal project neurons; CPNs = callosal projection neurons; PNs = projection neurons. **(F)** UMAP plots showing the clustering of different cell populations and clustering by sample types for the merged Seurat object after integrated ETV2plus NCs and NCs with the references databases from human brain organoids, vascularized human brain organoids, fetal and adult human brains.

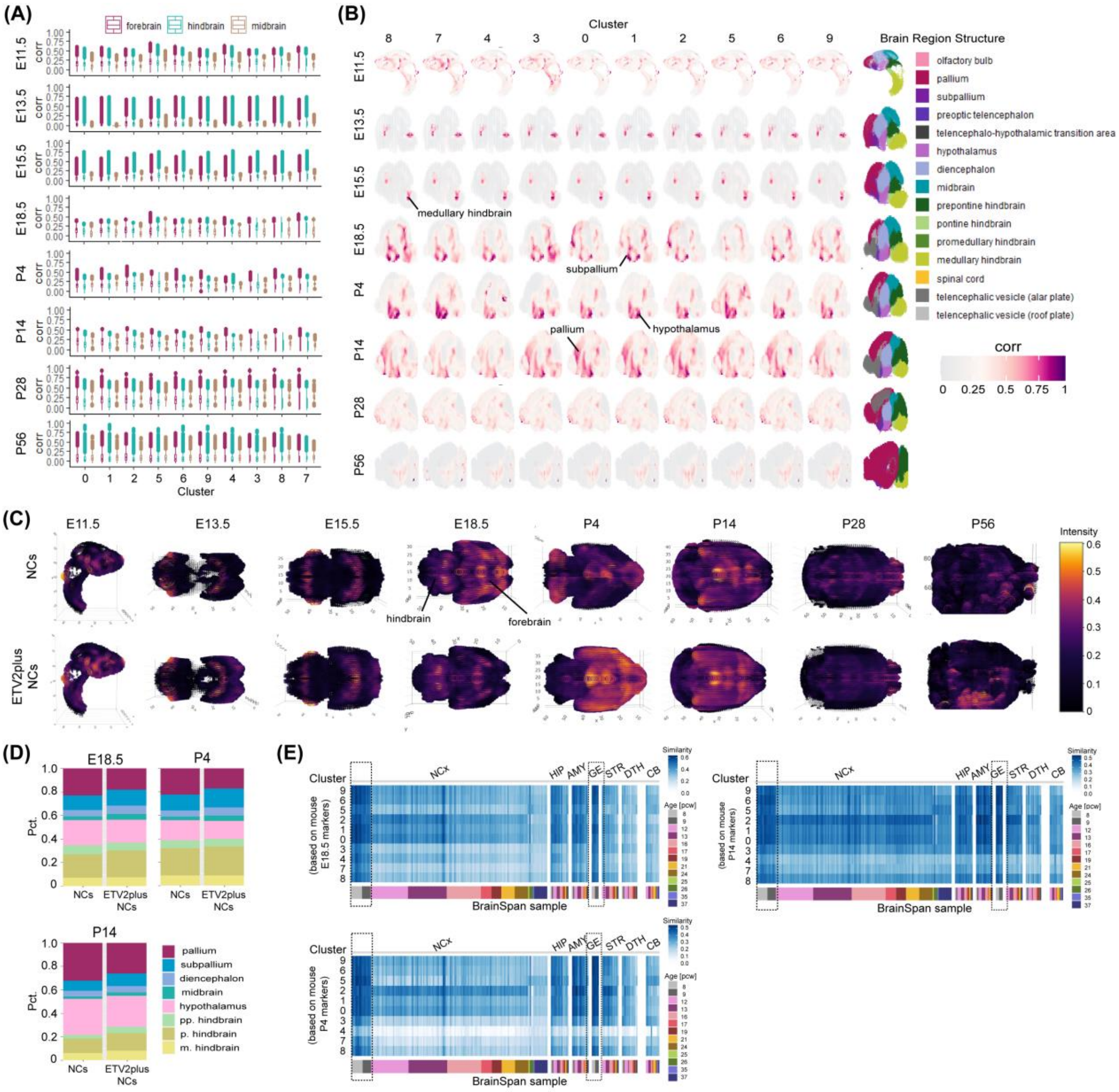

Figure S6: **(A)** Estimated correlation (corr) of each cluster identified in the merged Seurat object of ETV2plus NCs and NCs with the spatial expression of regional marker genes representing forebrain, hindbrain and midbrain at the different embryonic (E11.5, E13.5, E15.5 and E18.5) and postnatal (P4, P14, P28 and P56) stages of the mouse brain. Noted that in general the correlation with forebrain and hindbrain was higher than that of midbrain across the 10 cell clusters. The correlation was less variable (indicated by the less spreading of the data points of the bar plots) at E18.5, P4 and P14 indicating higher correlation than other stages. **(B)** 2D cross-sectional spatial mapping of the correlation of regional marker genes across the 10 cell clusters to that of the developing mouse brain at different stages. **(C)** Computed 3D mouse brain models (top view; left to right = hindbrain to forebrain) showing *in silico* spatial mapping of the expression level (by intensity) of top-10 regional marker genes by ETV2plus NCs and NCs to that of the developing mouse brain at different stages. **(D)** Regional composition analysis for

the expression level (by intensity) of top-10 regional marker genes by ETV2plus NCs and NCs to that of developing mouse brain at E18.5, P4 and P14 stages. pp. hindbrain = prepontine hindbrain; p. hindbrain = pontine hindbrain; m. hindbrain = medullary hindbrain. **(E)** Heatmap showing *in silico* similarity estimation of the transcriptomic profiles of each cluster identified across the 10 cell clusters to the human BrainSpan databases at different ages (by post-conception week; pcw), based on the top-10 mouse brain regional marker genes at E18.5, P4 and P14. NCx = neocortex; HIP = hippocampus; AMY = amygdala; GE = ganglionic eminences; STR = striatum; DTH = dorsal thalamus; CB = cerebellum.

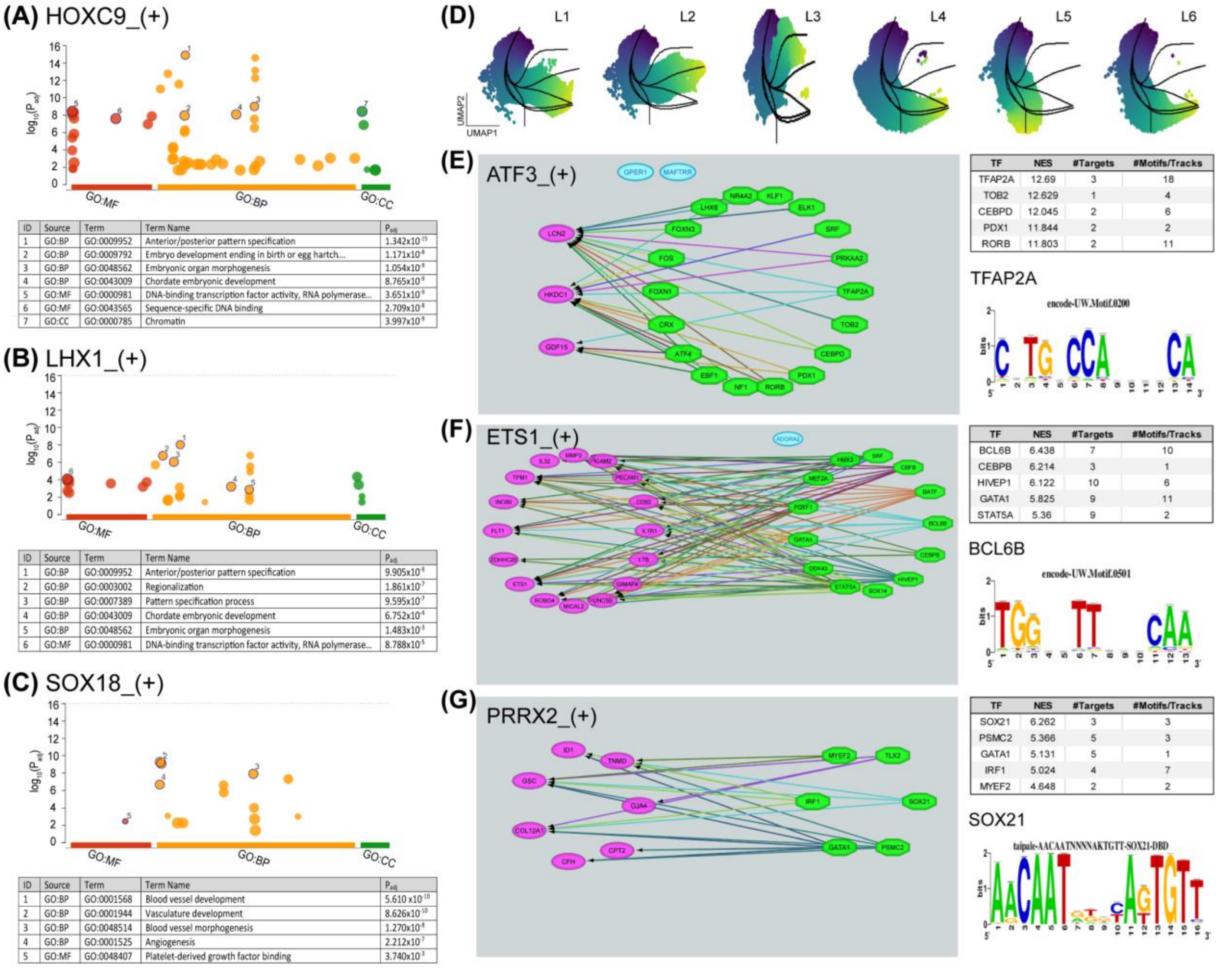

Figure S7: **(A, B, C)** The functionally enriched pathways associated to regulon *HOXC9_(+)*, *LHX1_(+)* and *SOX18_(+)* as identified via g:Profiler. **(D)** The heatmaps of the identified six lineage trajectories by *Slingshot* analysis. **(E, F, G)** The mapped gene regulatory networks for the identified top-3 regulons via iRegulon, and the predicted top-5 most enriched TFs (including the motif logo for the most enriched TFs), respectively*: ATF3_(+)*, *ETS1_(+)*, and *PRRX2_(+)*. NES = normalized enrichment score; Green nodes = TFs; Purple nodes = target genes; Blue nodes = genes identified in pySCENIC but not in iRegulon analysis.

Extended Description of Methods:

(1) Preparation of Neural Maintenance Medium (NMM)

Neural maintenance medium was prepared by mixing N2 medium and B27 medium as shown in table below at 1:1 ratio. The mixed medium was sterilized using 0.22 µm Bottle Top Vacuum Filter (Corning) and stored at 4 ^{o}C up to maximum 3 weeks.

N2 medium	B27 medium
500 ml DMEM:F12 Glutamax; *Life Technologies*	500 ml Neurobasal medium; *Life technologies*
5 ml N2 supplement; *Life Technologies*	10 ml B27 supplement; *Life Technologies*
250 µl Insulin; *Sigma*	5 ml Glutamax; *Life Technologies*
1 ml of 2-mercaptoethanol 50mM; *Life Technologies*	5 ml Pen/Strep; *Sigma*
5 ml of MEM NEAA; *Life Technologies*	
5ml sodium pyruvate; *Life Technologies*	

(2) Embedding of NC and eNC in matrigel droplets with/without iETV2-ECs

The BD Matrigel™ hESC-qualified Matrix (BD Biosciences; 1:200 dilution) was thawed on ice to prevent gelation at room temperature. In the laminar flow, a sterile Parafilm was placed on a holder obtained from 200 ul pipette tips box, and concave cavities on Parafilm were created by gently pressing the Parafilm against the holes of the holder. Each NC and eNC were placed into the concave cavities individually, and 35 ul of pre-cooled matrigel resuspended with or without 1 million day 8 iETV2-ECs was added to each NC and eNC. The matrigel-embedded samples were then incubated for 30 minutes in a cell culture incubator at 37 ^{o}C and 95% humidity to allow gelation. Subsequently, the matrigel droplets were transferred into NMM supplemented with BDNF, NT3 and bFGF for 3 days of static culture, after which they were transferred onto a shaker for dynamic culture in BDNF and NT3-supplemented NMM up to day 70.
